# Discovery of a polymorphic gene fusion via bottom-up chimeric RNA prediction

**DOI:** 10.1093/nar/gkae258

**Published:** 2024-04-08

**Authors:** Justin Elfman, Lynette Goins, Tessa Heller, Sandeep Singh, Yuh-Hwa Wang, Hui Li

**Affiliations:** Department of Biochemistry and Molecular Genetics, University of Virginia, Charlottesville, VA 22903, USA; Department of Biological Sciences, Clemson University, Clemson, SC 29631, USA; Department of Biochemistry and Molecular Genetics, University of Virginia, Charlottesville, VA 22903, USA; Department of Biochemistry and Molecular Genetics, University of Virginia, Charlottesville, VA 22903, USA; Computational Toxicology Facility, CSIR-Indian Institute of Toxicology Research, Lucknow, 226001, Uttar Pradesh, India; Department of Biochemistry and Molecular Genetics, University of Virginia, Charlottesville, VA 22903, USA; Department of Biochemistry and Molecular Genetics, University of Virginia, Charlottesville, VA 22903, USA; Department of Pathology, University of Virginia, Charlottesville, VA 22903, USA

## Abstract

Gene fusions and their chimeric products are commonly linked with cancer. However, recent studies have found chimeric transcripts in non-cancer tissues and cell lines. Large-scale efforts to annotate structural variations have identified gene fusions capable of generating chimeric transcripts even in normal tissues. In this study, we present a bottom-up approach targeting population-specific chimeric RNAs, identifying 58 such instances in the GTEx cohort, including notable cases such as *SUZ12P1*–*CRLF3*, *TFG*–*ADGRG7* and *TRPM4*–*PPFIA3*, which possess distinct patterns across different ancestry groups. We provide direct evidence for an additional 29 polymorphic chimeric RNAs with associated structural variants, revealing 13 novel rare structural variants. Additionally, we utilize the All of Us dataset and a large cohort of clinical samples to characterize the association of the *SUZ12P1*–*CRLF3*-causing variant with patient phenotypes. Our study showcases *SUZ12P1*–*CRLF3* as a representative example, illustrating the identification of elusive structural variants by focusing on those producing population-specific fusion transcripts.

## Graphical Abstract

**Graphical Abstract:**
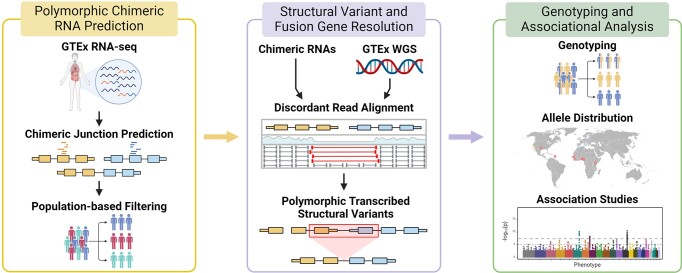

## Introduction

Chimeric RNAs, traditionally associated with gene fusion events, have long served as crucial markers for differentiating cancers and cell types. These unique RNA structures, along with the proteins they encode, have been instrumental in understanding and diagnosing various diseases. However, recent research has unraveled a new layer of complexity: chimeric RNAs can emerge from intergenic splicing, independent of gene fusion events (1,2). Some of the most impactful findings in this field have stemmed from the discovery of chimeras such as *JJAZ1*–*JAZF1* (3,4), *PAX3*–*FOXO1* (5) and more recently *EML4*–*ALK* (6), which mirror known oncogenic gene fusions in the absence of their associated changes at the DNA level. These findings have been further supplemented by read-through chimeras such as *SLC45A3*–*ELK4* (7) and chimeras such as *ASTN2*–*PAPPA_AS_* (8), which are produced without noted changes to subject DNA and play roles relevant to the development of cancer. To uncover more of these intriguing RNA structures, substantial efforts have been invested in cataloging chimeric RNAs across various diseases and cell types. This ongoing exploration aims to identify novel chimeras and expand our understanding of their roles in health and disease (9–16).

In parallel to our examination of chimeric RNAs that result from splicing phenomena at the RNA level, there is a growing body of research that aims to characterize structural variation genome-wide (17). Detecting structural variants (SVs) poses a significant challenge, particularly in repetitive regions and when annotating complex variants and inversions. Recent initiatives have aimed to tackle these complexities by examining a large pool of genomes simultaneously (18) and meticulously scrutinizing a few genomes using a combination of short- and long-read sequencing (19).

This line of research forms a valuable foundation for subsequent analysis, and the integration of SV annotation with biological data is anticipated to play a pivotal role in understanding the significance of these variations. Recent studies have linked structural variation with gene expression in healthy individuals from the GTEx cohort, demonstrating especially strong effects from variants that affect annotated genes (20,21). While identification of SVs that produce gene fusions is relatively rare in these studies, they stand out as noteworthy discoveries more likely to result in functional products (18).

Several methods have been developed to harness transcriptomic data for predicting SVs (22–24). Some specifically focus on chimeric transcript prediction to predict gene fusion events (25–27). Collectively, these methods can be classified as bottom-up approaches, where they identify evidence of the transcript and seek to trace it back to the underlying DNA-level change. While bottom-up approaches miss fusions that are not transcribed in the test model, they enrich for gene fusions that affirmatively produce transcripts.

In this study, we showcase the effectiveness of a bottom-up approach in identifying polymorphic SVs by targeting chimeric RNAs that exhibit population-specific patterns. This prediction not only provides value for genotyping and population stratification, but also specifically filters for variants that affect parental genes and produce chimeric RNAs. In this report, we discover 58 population-specific chimeric RNAs and present a comprehensive characterization of *SUZ12P1*–*CRLF3* as a representative polymorphic chimeric RNA. We investigate its prevalence across global populations, provide exemplary characterization of this fusion event and its transcript, and present direct evidence for an additional 31 polymorphic chimeric RNAs, including 13 novel rare SVs that produce fusion transcripts.

## Materials and methods

### Data acquisition

RNA sequencing (RNA-seq) and corresponding whole-genome sequencing (WGS) data were obtained from the GTEx project (V6 dbGaP accession phs000424.v6.p1) and the Geuvadis Consortium repository via the 1000 Genomes data hub. Expression and sample data for GTEx samples were also obtained from the GTEx project (V6 dbGaP accession phs000424.v6.p1).

All of Us SV annotations were obtained from the short-read WGS (srWGS) SV VCF, and variants corresponding to those producing *SUZ12P1*–*CRLF3*, *TFG*–*ADGRG7* and *TRPM4*–*PPFIA3* were selected based on exact matching of chromosome, start position and SV type. All of Us genetic-predicted ancestry inferences were obtained from the ancestry_preds.tsv auxiliary file.

### Polymorphic chimeric RNA predictions

Chimeric RNAs were predicted according to a previous publication (9). Briefly, chimeric RNA predictions were generated using the default parameters of EricScript (28), and using GRCh38 as reference. We filtered out the following chimeric RNAs with low confidence: (i) chimeric RNAs with a prediction score (EricScore) <0.6; (ii) chimeric RNAs without clear breakpoint positions; and (iii) chimeric RNAs that are highly similar to existing annotated transcripts (>90% identity). Exon boundary designations and fusion protein frame prediction were also generated according to a previous publication (9).

In order to enrich for polymorphic chimeric RNAs, we selected chimeric RNAs that were detected in <250 unique individuals within the GTEx cohort, and within each individual, the chimeric RNA was expressed in >5 unique tissues in >66% of samples.

### PCR of chimeric RNAs

Clinical human leukocytes were obtained in accordance with the Institutional Review Board within the University of Virginia Health System. Whole blood samples were collected from patients admitted to the hospital for non-cancer ailments, and leukocytes were enriched by centrifugation. RNA was extracted using standard protocol for the TRIzol reagent, and reverse transcription was conducted using the standard protocol for the SensiFAST cDNA Synthesis Kit (Bioline, BIO-65054). Quantitative polymerase chain reaction (qPCR) was conducted using the StepOne Plus System (Life Technologies) using the standard protocol for SensiFAST SYBR w/ Hi-Rox (Bioline, BIO-92005). Following reverse transcription qPCR (RT-PCR) and gel electrophoresis, DNA was extracted using the standard protocol for the PureLink Gel Extraction Kit (Invitrogen, K210012) and sent to Genewiz for Sanger sequencing.

Patient autopsy samples were obtained from the University of Virginia Health System under Institutional Review Board protocol. Complementary DNA was produced via the same methodology detailed above, and PCR was conducted using the standard protocol for Bioline MyTaq™ Red Mix (BIO-25043).

### PCR genotyping of leukocytes

PCR genotyping was performed utilizing single-nucleotide polymorphisms (SNPs) rs16891982 and rs1426654. rs16891982_G and rs1426654_A were considered to correspond to the European grouping, while rs16891982_C and rs1426654_G were considered to correspond to the non-European grouping. We ordered Thermo Fisher Scientific probes C_2842665_10 and C_2908190_10, and performed the assay with the Bioline SensiFAST SYBR Hi-Rox Kit (BIO-92005) per standard protocol. In order to assign a genotype, we required the result to exhibit a standard amplification curve and be consistent across all replicates. Additionally, samples were designated European or non-European only if the designated genotypes for both alleles were in agreement. Otherwise, samples were labeled ‘unknown’.

PCR genotyping targeting the *SUZ12P1*–*CRLF3* rearrangement was conducted on all samples identified as positive by the TaqMan genotyping assay, as well as 10 samples identified as negative for the rearrangement. We designed four primers to amplify around breakpoints on either side of the inversion (Figure 4D and E) and used the standard protocol for Bioline MyTaq™ Red Mix (BIO-25043) for amplification.

Primers used in this study are provided in Supplementary Table S1.

### *In silico* AGREP genotyping

The base AGREP function (29) was used to compare a set of query sequences against quality-filtered fastq files. Reads identified to match the query sequences were then aligned with pBLAT (30,31) using parameters minMatch = 1, minScore = 90 and minIdentity = 90, and filtered using default parameters of the BLAT suite function repsPSL. These outputs were compared to a 100-kb region surrounding the region of interest. Hits were designated true positive if they mapped exclusively to the locus, false positive if mapped exclusively to other loci and uncertain if the read mapped to multiple different loci, as long as one was the target locus. All counts indicated within this report are true positive only. Seventy base pair query sequences were designed to map onto each of three regions of interest: the region spanning breakpoint 1 (SC_Ctrl_); within the deleted region of the breakpoint (SC_Del_); and spanning breakpoints 2 and 3 (SC_Inv_) (Supplementary Figure S1). Samples were noted as SC+ if SC_Ctrl_ and SC_Inv_ were present, and SC_Del_ was absent. The TFG–ADGRG7 variant was genotyped with a 55-bp sequence crossing the intronic junction, and the TRPM4–PPFIA3 variant was genotyped with a 70-bp sequence crossing the intronic junction (Supplementary Table S5).

### Read alignment and visualization

Reads were quality filtered using default parameters of the NGSQC toolkit (32), and aligned to GRCh38 with BWA (33). Split and discordant reads were extracted and selected by genomic region using Samtools (33), and visualized based on pair orientation in Integrative Genomics Viewer (IGV) (34).

### Secondary structure prediction

We performed predictions centered on breakpoint 1 (chr17:30770904), breakpoint 2 (chr17:30775728) and breakpoint 3 (chr17:30778956) using the methodology described by Szlachta *et al.* (35) using the ViennaRNA package 2.0 (36). Two sets of parameters were considered: predictions within ±1000 nt with a 300-nt window and 150-nt step and ±200 nt with a 30-nt window and 1-nt step. Additionally, we performed prediction using both positive and negative strands surrounding rs145766379.

### PheWAS

All of Us sample cohorts were selected to include donors who had srWGS data, corresponding SV annotation data, and at least one entry under disease, neurofibromatosis type 1 (NF1) and acute cerebrovascular disease (ACD) conditions (concept IDs 381591, 4274025 and 377252). Patient data including date of birth and sex at birth were also pulled and incorporated into the dataset. Age was derived as the number of days between subject date of birth and current date of the analysis divided by 365, rounded to the nearest year. SNOMED codes associated with patient conditions were converted into phecodes using PheMap (37), and filtered to remove duplicate collapsed phecode entries per donor. Donors were genotyped according to their SV annotation, using either a binary identifier or allele count depending on the disease model assumption to be run.

PheWAS was run using the R PheWAS package (38) based on genotyping on SUZ12P1–CRLF3, TFG–ADGRG7 and TRPM4–PPFIA3 variants, assuming (i) a dominant disease model (binary genotype, logistic regression) or (ii) an additive disease model (allele count, linear regression), and in (a) all included donors or (b) donors of the most common predicted ancestry for each variant. In all iterations, age, sex at birth and the first five ancestry principal components were used as covariates in analysis.

### Other biostatistics

**Figure 1.**
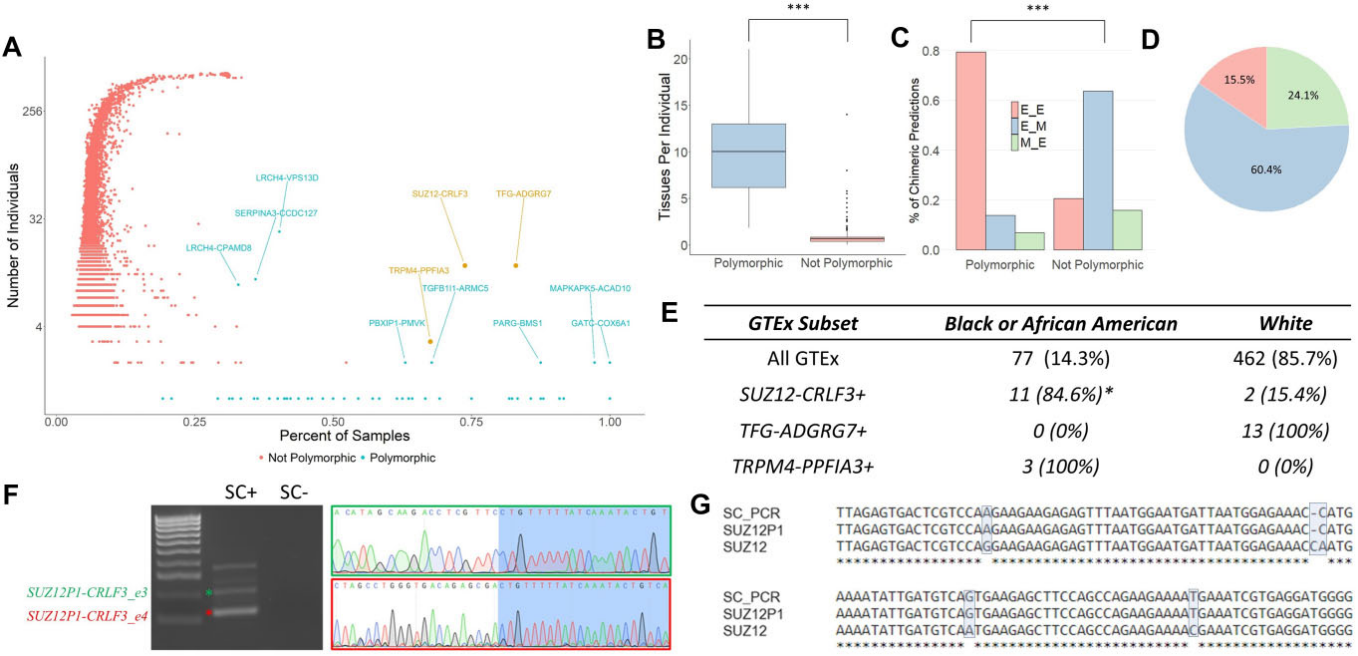
Identification and validation of the *SUZ12P1*–*CRLF3* chimeric RNA. (**A**) GTEx chimeric RNAs by the number of unique individuals in which they are found and the mean proportion of samples for each individual in which they are found. Chimeric RNAs in green have been selected by the polymorphic filter, and those in red have been excluded. *SUZ12*–*CRLF3*, *TFG*–*ADGRG7* and *TRPM4*–*PPFIA3* are further highlighted in gold. (**B**) Comparison of the average number of tissues each chimeric RNA is found per individual, grouped by polymorphic filter (*P* = 2.2 × 10^−16^). (**C**) Exon junctions for each chimeric prediction (*P* = 2.2 × 10^−16^). End-of-exon (E) junctions indicate a match to an annotated exon breakpoint ± 2 bp, while middle-of-exon (M) junctions indicate non-end-of-exon designations. (**D**) Potential protein frame predictions for polymorphic-filtered chimeric RNAs. (**E**) Racial demographics of the GTEx cohort and subsets of the GTEx cohort, including those who express *SUZ12P1*–*CRLF3*, *TFG*–*ADGRG7* and *TRPM4*–*PPFIA3*. (**F**) RT-PCR and Sanger sequencing supporting the validation of two *SUZ12P1*–*CRLF3* isoforms (asterisks). (**G**) Transcript sequence confirms that the *SUZ12P1–CRLF3* chimeric RNA retains sequence from *SUZ12P1* rather than *SUZ12*.

Comparison of means between polymorphic and non-polymorphic groups in Figure 1B was performed via Student’s *t*-test. Comparison of exon junction designations in Figure 1C was performed via a chi-square test of independence on count data, and presented as percentages within each respective population.

Comparisons between SC+ and SC− expression groups were performed via Student’s *t*-test, assuming equal variance except in cases where variance was found to be unequal. Overrepresentation of clinical codes between SC+ and SC− groups was performed via logistic regression, utilizing all clinical codes with at least 20 total instances, including at least 1 instance in the experimental group. Gender, genotype-corrected inferred ancestry and age were included in the model as covariates. Odds ratio (OR) was calculated as the natural exponent of the log odds coefficient. Bonferroni correction was included for all tests where multiple testing correction is applicable, including PheWAS and clinical code overrepresentation. A *P*-value cutoff of 0.05 was used to determine statistical significance.

## Results

### Identification and validation of polymorphic chimeric RNAs

#### De novo prediction and polymorphic filtering of chimeric RNAs

To predict polymorphic chimeric RNAs, we harnessed a dataset of 9496 RNA-seq samples from the GTEx cohort (V6), spanning 53 diverse tissues from 549 unique donors. Employing our chimeric prediction pipeline (39), we identified a total of 14 768 potential chimeric RNAs. To focus on polymorphic patterns, we refined our selection by filtering for chimeras predicted in <250 individuals, yet present in >5 unique tissues in 66% of individuals expressing the chimera, aiming to capture transcripts with widespread occurrence but restricted to specific populations (Figure 1A and Supplementary Table S2). These criteria resulted in 58 predictions, markedly distinct from the remaining 14 711 predictions, which can be visualized by the average number of tissues in which each is found in per individual (Figure 1B).

Putative polymorphic predictions were highly enriched in annotated end-of-exon to end-of-exon splice junctions (E_E), a robust indicator of prediction accuracy based on our experience (Figure 1C). Additionally, many polymorphic chimeras were predicted to potentially form fusion proteins, with 15.5% retaining the parental reading frame and 24.1% generating frameshifted peptides (Figure 1D).

#### Characterization of ancestry-specific chimeric RNAs

In our exploration of ancestry-specific chimeric RNAs, three distinct chimeric RNAs, *SUZ12*–*CRLF3*, *TFG*–*ADGRG7* and *TRPM4*–*PPFIA3*, were observed to exhibit stratification based on the reported race of donors (Figure 1E). Of these, *SUZ12*–*CRLF3* displayed a statistically significant difference, and was found to be enriched in black or African American donors. Though not statistically significant in this study, *TFG*–*ADGRG7* has been previously reported as a chimeric RNA specific to European ancestry (40). *TRPM4*–*PPFIA3* was identified in too few samples to draw statistically significant conclusions. Through PCR validation of *SUZ12*–*CRLF3*, we discovered that the fusion transcript is not formed by the *SUZ12* polycomb gene, but rather by its pseudogene, *SUZ12P1*, in conjunction with *CRLF3*. We identified two isoforms of this transcript, with junction sequences formed by the joining of *SUZ12P1* exon 7 to *CRLF3* exon 3 or 4 (Figure 1F and G).

To validate these findings in another independent dataset, we extended our analysis to the 1000 Genomes cohort, which includes RNA-seq data in a subset of samples (41). We directly compared 28 base pairs spanning the junction sequence of each isoform of the predicted *SUZ12P1*–*CRLF3* chimera to quality-filtered RNA-seq reads (Supplementary Figure S1). We observed *SUZ12P1*–*CRLF3* in the Nigerian population (YRI) at a similar rate to what was found in African American GTEx samples, while it was absent in any of the four European populations (Table 1). This cross-population validation strengthens the evidence for ancestry-specific expression of the *SUZ12P1*–*CRLF3* chimeric RNA.

**Table 1.** SUZ12P1–CRLF3 occurrence in 1000 Genomes and GTEx populations

Cohort	Description	Detected	Total	Percentage
CEU	Northern European (Utah)	0	92	0
FIN	Finnish in Finland	0	95	0
GBR	British in England and Scotland	0	95	0
TSI	Toscani (Italy)	0	93	0
YRI	Yoruba in Ibadan (Nigeria)	13	89	14.6
GTEx	White	2	462	0.4
GTEx	Black or African American	11	77	14.3
GTEx	Other/unknown	0	10	0

#### Validation and enumeration of the SUZ12P1–CRLF3 chimeric RNA in clinical blood samples

**Figure 2.**
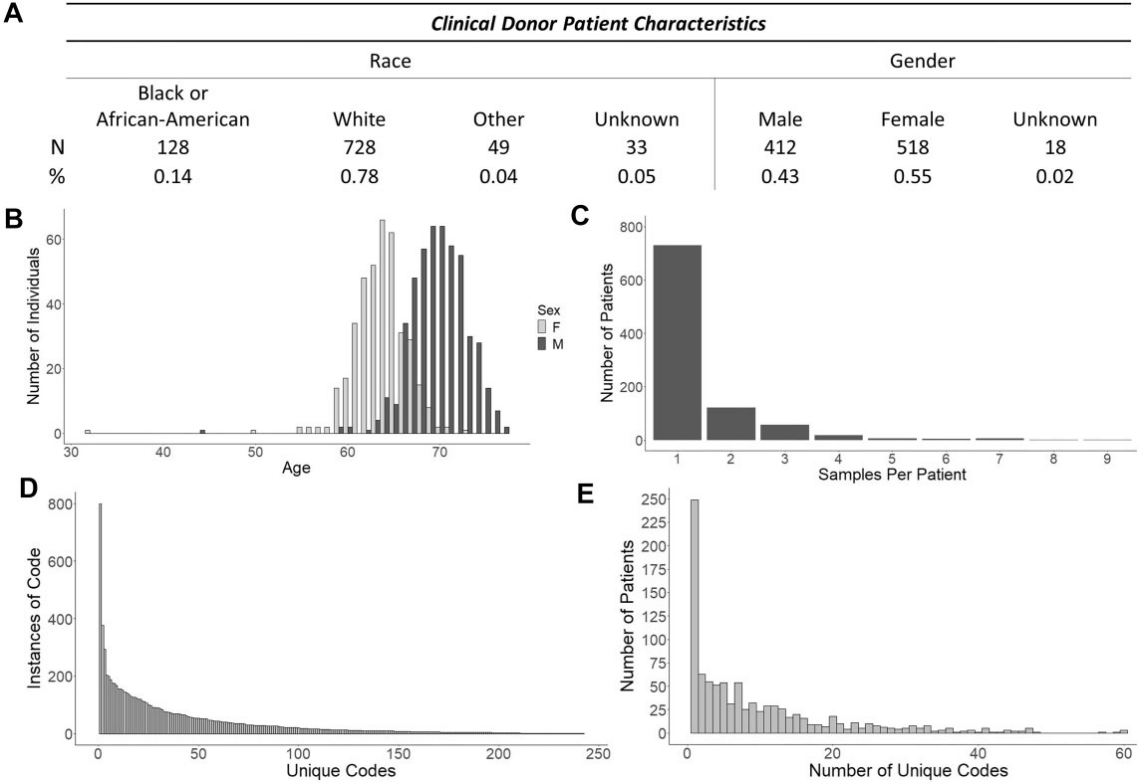
One thousand three hundred fifty-one clinical donor leukocytes collected from the University of Virginia Health System. (**A**) Recorded clinical donor patient characteristics. (**B**) Age distribution of the dataset, by listed sex. (**C**) Number of samples per unique patient. (**D**) The number of recurrences per unique clinical code. (**E**) The total number of clinical codes exhibited per patient.

To validate and quantify the presence of the SUZ12P1–CRLF3 chimeric RNA, we extended our analysis to a collection of clinical leukocyte samples obtained from patients admitted for non-cancer conditions by the University of Virginia Health System. This dataset encompasses 1351 total samples from 948 unique donors. Reflecting the patient population, this cohort is predominantly male and white (Figure 2A), and the mean age of male donors significantly exceeds the mean age of female donors (Figure 2B). Of the 948 donors, 219 have provided samples at multiple time points (Figure 2C). Additionally, clinical codes are reported for these donors, providing insights into the clinical phenotypes of donors. We find that the median code is listed in 24 unique instances and the 24 most common codes account for approximately half of all annotated codes (Figure 2D), and the median donor possesses six unique codes (Figure 2E).

**Figure 3.**
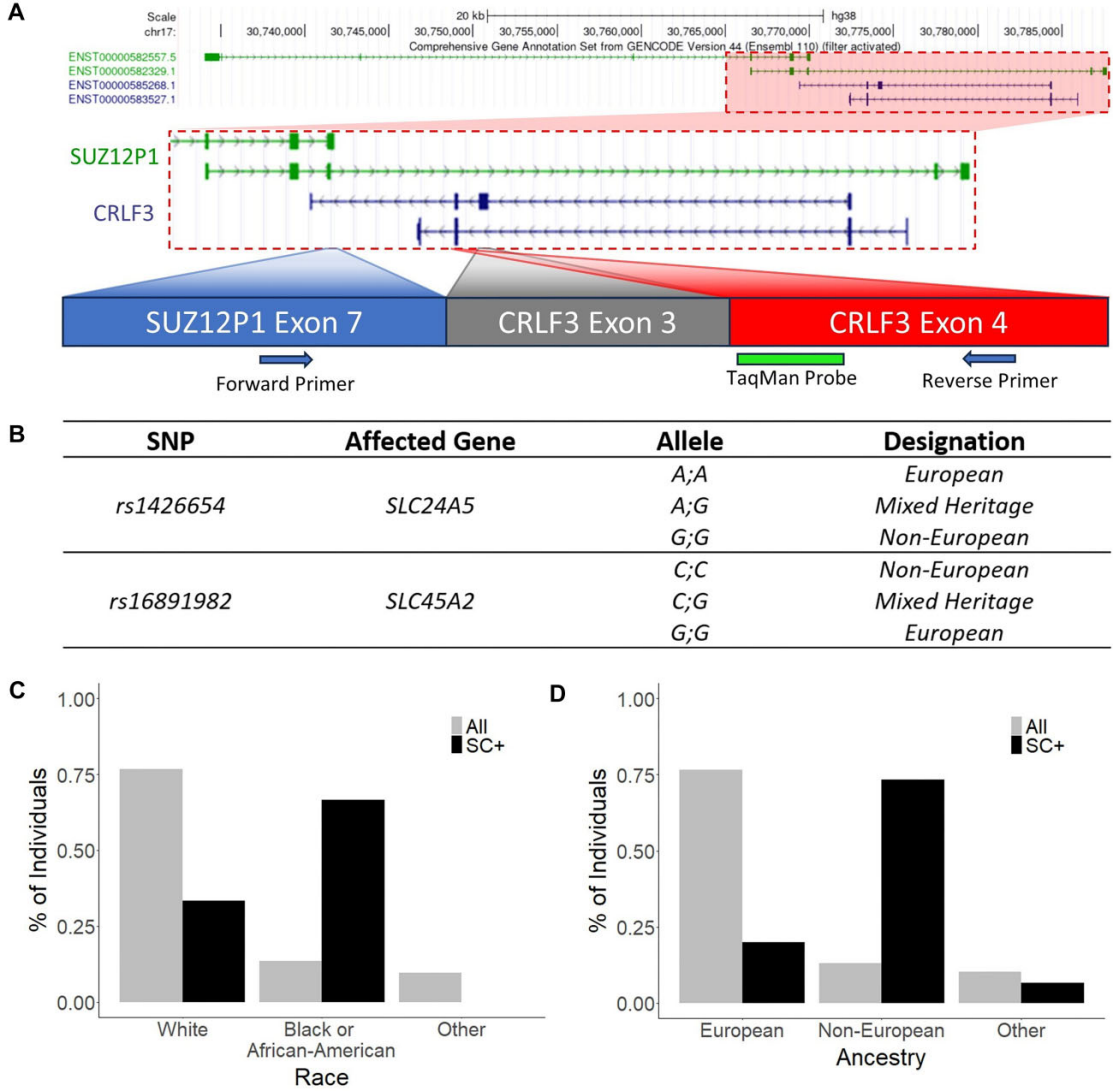
Characterization of *SUZ12P1*–*CRLF3* in clinical samples. (**A**) Probe-based qPCR design and contextualization of overlapping *SUZ12P1* and *CRLF3* isoforms. (**B**) SNPs used to genotype leukocyte donors paired with their heritage designation. Proportion of population expressing the *SUZ12P1*–*CRLF3* chimeric RNA grouped by reported race compared to the proportion of the population classified by (**C**) annotated race prior to SNP genotyping and (**D**) annotated heritage following SNP genotyping.

We designed a TaqMan qPCR assay to detect both isoforms of *SUZ12P1*–*CRLF3* within this patient population (Figure 3A). Given that clinical racial classifiers do not accurately represent ancestry, we refined our stratification using ancestry-informative markers, rs16891982 and rs1426654, which correlate strongly with ancestral population (42). Donors were genotyped using these SNPs, and designated accordingly as European, mixed heritage or non-European (Figure 3B). We utilized TaqMan SNP genotyping assay probes to test all samples expressing *SUZ12P1*–*CRLF3*, as well as a panel of both white and African American individuals not expressing *SUZ12P1*–*CRLF3*. In all cases where sample genotyping was successful, the population designations for rs1426654 and rs16891982 were in agreement, and of these, the sample designation was changed in six cases. Notably, five cases previously labeled as unknown race were reclassified as non-European, while one case originally designated as white was reclassified as non-European (Supplementary Figure S2).

Prior to SNP genotyping correction, *SUZ12P1*–*CRLF3* was expressed significantly more frequently in patients reported as black or African American compared to those reported as white or other (Figure 3C). However, the proportion of individuals categorized as black or African American was considerably lower than that observed in the GTEx and 1000 Genomes cohorts. After SNP genotyping correction, donors expressing *SUZ12P1*–*CRLF3* were more likely to possess genetic heritage from non-European populations, approaching a similar proportion (10.1%) to that observed in the GTEx African American and 1000 Genomes YRI populations (∼14.5%) (Figure 3D).

### The *SUZ12P1*–*CRLF3* RNA is generated by a genomic rearrangement at 17q11.2

#### The SUZ12P1–CRLF3 transcript is found in multiple tissues of a SUZ12P1–CRLF3-positive donor

**Figure 4.**
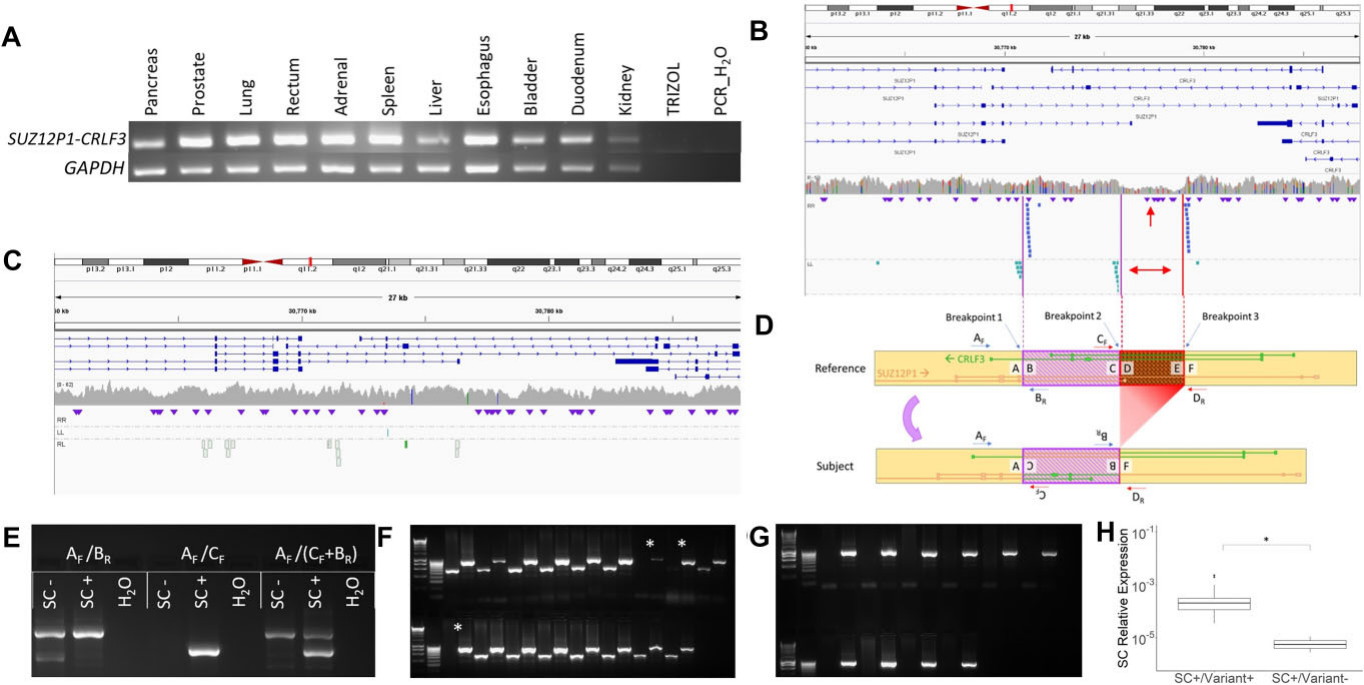
Characterization of the *SUZ12P1–CRLF3* chromosomal rearrangement. (**A**) The *SUZ12P1*–*CRLF3* chimeric RNA is detected in all provided tissues of a single donor. (**B**) Discordant read alignment reveals an inversion and adjacent deletion at the *SUZ12P1*–*CRLF3* locus in an individual expressing the chimeric RNA, and (**C**) these are absent in an individual who does not express the chimeric RNA. (**D**) Schematic of the *SUZ12P1*–*CRLF3* chromosomal rearrangement, representation of the chimeric RNA and primer design for genotyping PCR. (**E**) Multiplexed genotyping PCR design. (**F**) Genotyping assay applied to 17 *SUZ12P1*–*CRLF3*-expressing samples. Asterisks denote *SUZ12P1*–CRLF3-expressing samples without the *SUZ12P1–CRLF3*variant. (**G**) Genotyping assay applied to 10 non-*SUZ12P1*–*CRLF3*-expressing samples. (**H**) *SUZ12P1*–*CRLF3* expression relative to GAPDH, grouped by SUZ12P1–CRLF3 variant genotyping. The three samples denoted with an asterisk in panel (F) comprise the Variant− group.

In the GTEx dataset, the *SUZ12P1*–*CRLF3* transcript was found in multiple tissues of each expressing donor. We sought to confirm this observation experimentally. We obtained autopsy samples from 13 unique donors with a recorded race of black or African American within the University of Virginia Health System, and found that one individual expressed *SUZ12P1*–*CRLF3*. We then obtained samples from 11 different tissues from this *SUZ12P1*–*CRLF3*-positive donor, and found the RNA expressed ubiquitously across tissues (Figure 4A), suggesting that the fusion transcript may result from a polymorphism.

#### Discordant read alignment reveals a complex rearrangement at the SUZ12P1–CRLF3 locus

*SUZ12P1* and *CRLF3* are situated in opposing orientations on the same locus within 17q11.2, with several isoforms overlapping coordinates. This proximity, coupled with the consistent rate of detection, population specificity and lack of tissue specificity, formed a strong basis that the *SUZ12P1*–*CRLF3* chimeric RNA may be generated by a polymorphic rearrangement. In order to confirm this hypothesis, we extracted split and discordant reads from all 13 GTEx samples expressing *SUZ12P1*–*CRLF3* as well as 11 random GTEx samples not expressing *SUZ12P1*–*CRLF3*.

Alignment of these reads to HG38 revealed a pile-up within a region on 17q11.2, overlapping isoforms of both *SUZ12P1* and *CRLF3*. The alignment of the left–left and right–right reads indicated an inversion, and the spacing of the right–right reads around the downstream inversion breakpoint, as well as the depletion of reads aligning to this region, suggested a deletion in the subject’s genome (Figure 4B). Neither of these observations are present in the genome of a *SUZ12P1*–*CRLF3−* individual (Figure 4C). Figure 4D provides a graphical representation of this rearrangement, illustrating the inversion in purple and the adjacent deletion in red. Two different isoforms of *SUZ12P1*–*CRLF3* are generated within the inversion, as visualized on the subject allele.

#### Genotyping for the SUZ12P1–CRLF3 rearrangement

To experimentally confirm the SV at the DNA level, we developed a PCR genotyping assay to amplify over each breakpoint for both the reference and subject genomes (Figure 4D and E). This assay utilized the exchange in the orientation of primers B_R_ and C_F_ caused by the inversion. This assay was performed on the *SUZ12P1*–*CRLF3+*panel as well as a panel of samples from white donors not expressing *SUZ12P1*–*CRLF3*. Genotyping revealed that nearly all individuals expressing the *SUZ12P1*–*CRLF3* chimeric RNA were heterozygous for the alternate allele, whereas all *SUZ12P1*–*CRLF3*-negative cases had no amplification for the alternate allele (Figure 4F and G). Notably, three individuals expressing the *SUZ12P1*–*CRLF3* chimeric RNA did not possess evidence of the complex rearrangement. These individuals exhibited significantly lower expression than those possessing the transformed allele, indicating that the rearranged gene fusion confers enhanced expression of the fusion transcript (Figure 4H).

#### Bioinformatic evaluation of the SUZ12P1–CRLF3, TFG–ADGRG7 and TRPM4–PPFIA3 rearrangements

In our pursuit of broader insights, we established an *in silico* genotyping pipeline for WGS data. This pipeline, depicted in Supplementary Figure S1, leverages fuzzy string matching to identify unique genomic sequences and subsequently maps supporting reads back to the reference genome to confirm on-target findings. This computational approach enables us to extend our analysis to publicly available datasets, such as the 1000 Genomes Project and All of Us, and to use donor information for further associations.

**Figure 5.**
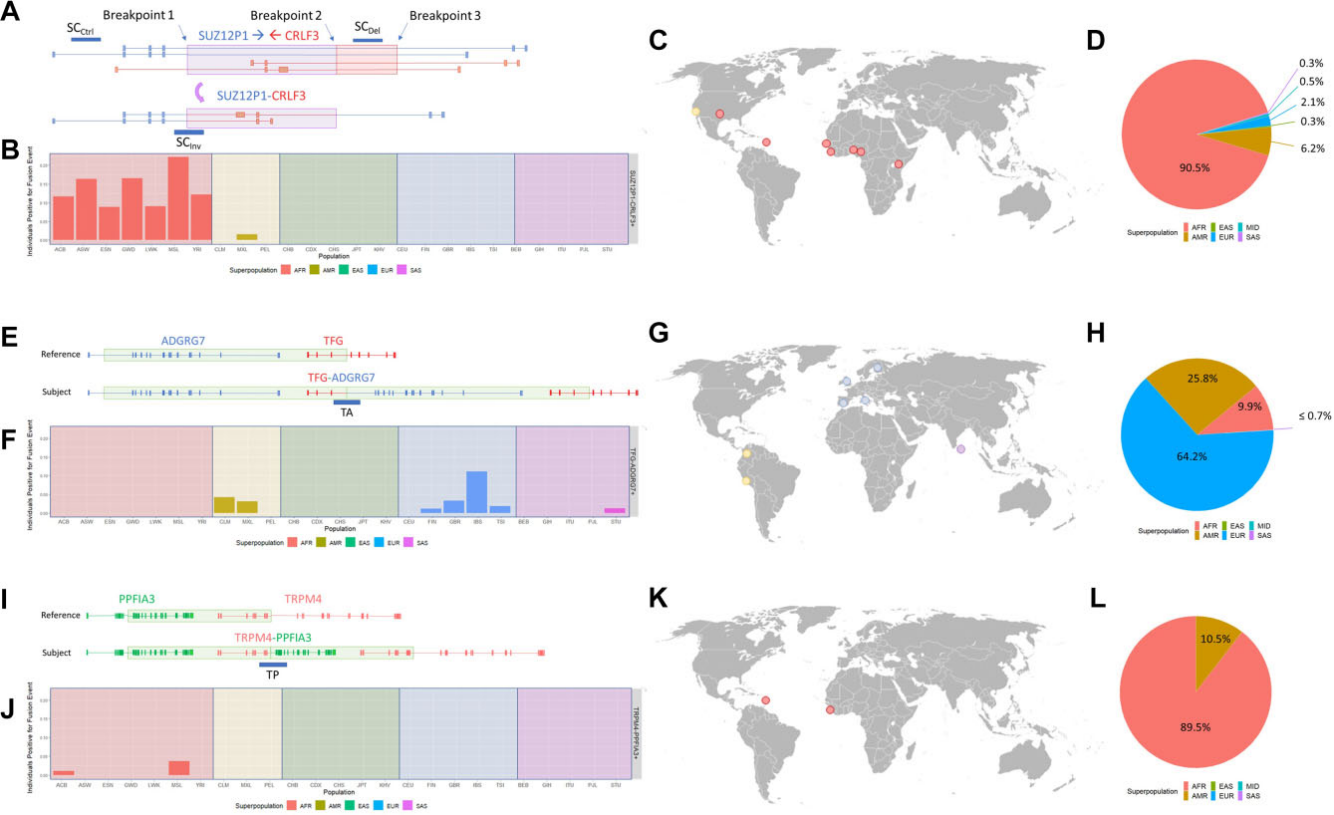
*In silico* genotyping of the 1000 Genomes cohort. (**A**) Representative graphic of the SUZ12P1–CRLF3 rearrangement and query locations for AGREP. (**B**) Frequency of variant allele detection by subpopulation and superpopulation for *SUZ12P1*–*CRLF3* and (**C**) distribution across the world map. (**D**) Proportions of inferred ancestry within SUZ12P1–CRLF3+ individuals within the All of Us Research Program. (**E**) Representative graphic of the TFG–ADGRG7 rearrangement and query location for AGREP. (**F**) Frequency of variant allele detection by subpopulation and superpopulation for *TFG*–*ADGRG7* and (**G**) distribution across the world map. (**H**) Proportions of inferred ancestry within TFG–ADGRG7+ individuals within the All of Us Research Program. (**I**) Representative graphic of the TRPM4–PPFIA3 rearrangement and query location for AGREP. (**J**) Frequency of variant allele detection by subpopulation and superpopulation for TRPM4–PPFIA3 and (**K**) distribution across the world map. (**L**) Proportions of inferred ancestry within TRPM4–PPFIA3+ individuals within the All of Us Research Program.

Applying the *in silico* genotyping pipeline to the 1000 Genomes cohort, we assessed the distribution of the SVs that produce *SUZ12P1*–*CRLF3* (Figure 5A–C), *TFG*–*ADGRG7* (Figure 5E–G) and *TRPM4*–*PPFIA3* (Figure 5I–K). The *SUZ12P1*–*CRLF3* variant was found exclusively in the African superpopulation, encompassing ACB, ASW, ESN, GWD, LWK, MSL and YRI subpopulations, with a single exception in the admixed MXL population (Figure 5B and C). Across all African subpopulations, minor allele frequencies were consistently above 5%, and allelic distribution was within the Hardy–Weinberg equilibrium (Supplementary Table S3). Additionally, the analysis of a sequence spanning the DNA junction of the *TFG*–*ADGRG7* fusion revealed its presence in European and admixed populations (Figure 5F and G), consistent with previous observation (40). Finally, the *TRPM4*–*PPFIA3* variant, a rare occurrence predicted in individuals with African ancestry, was exclusively identified in the African subpopulations (Figure 5J and K).

Similarly, we manually identified the matching SV annotation within the All of Us Research Program for the variants that produce *SUZ12P1*–*CRLF3* (CPX_chr17_116), *TFG*–*ADGRG7* rearrangement (DUP_chr3_2210) and the *TRPM4*–*PPFIA3* (DUP_chr19_2317) based on breakpoint and configuration. This information was then used to genotype donors within this cohort (Figure 5D, H and L). It is worth noting that this is an admixed American cohort, and superpopulation is inferred based on principal component analysis rather than donor selection. Except for an increased representation of the *TFG*–*ADGRG7* variant in the inferred African superpopulation, and all three variants in the inferred American superpopulation, each variant was predominantly ancestry-specific (Figure 5D, H and L).

### Implications of the *SUZ12P1–CRLF3* rearrangement

#### Absence of association between SUZ12P1–CRLF3 allele and parental gene expression and patient characteristics

**Figure 6.**
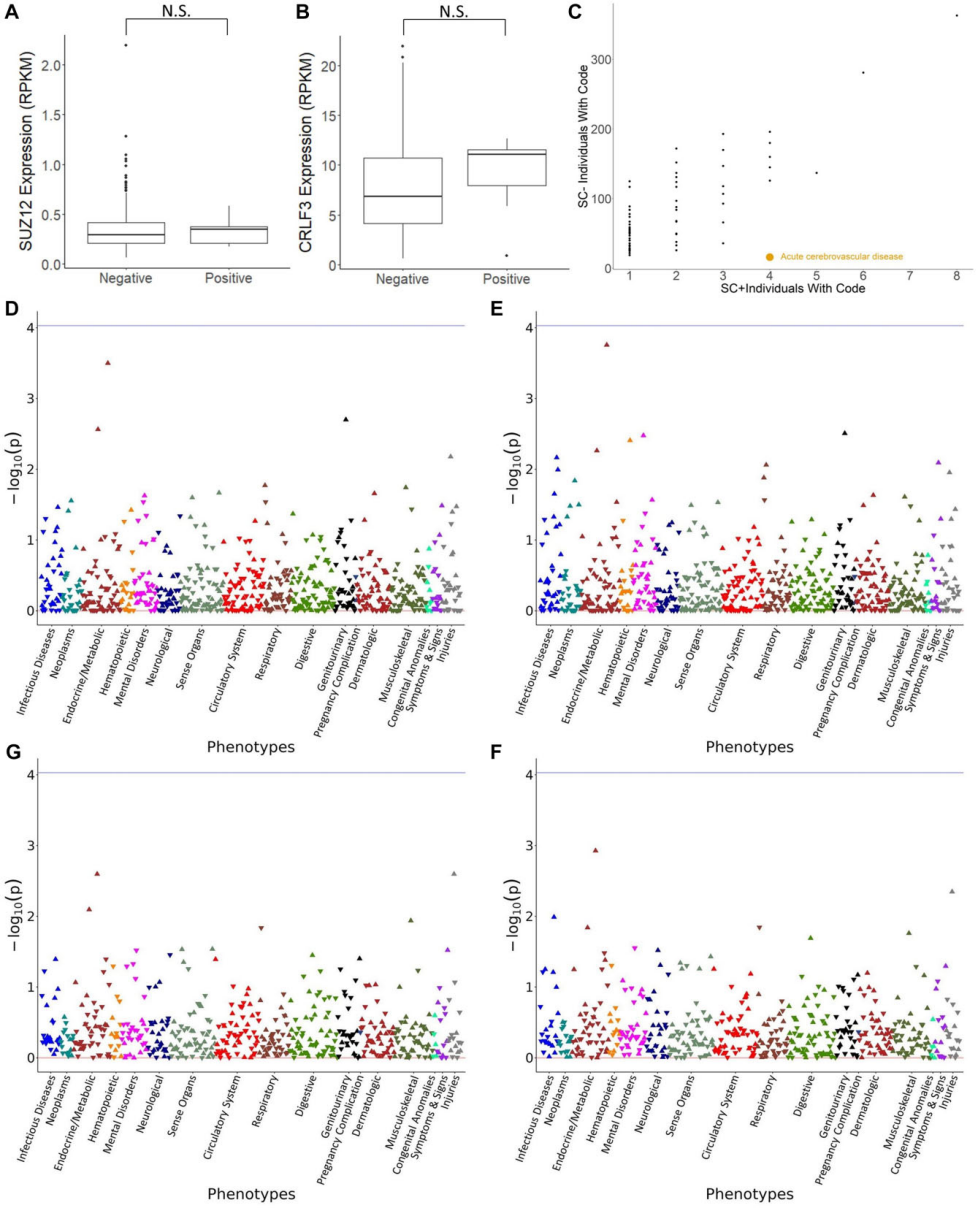
Functional implications of the *SUZ12P1–CRLF3* variant. (**A**) *SUZ12P1* and (**B**) *CRLF3* expression by rearrangement allele. (**C**) Clinical codes associated with leukocyte donors. The clinical code for ACD is highlighted in gold (*P* = 0.038, OR = 12.1). (**D**) Manhattan plot for the *SUZ12P1*–*CRLF3* PheWAS on All of Us cohort within the entire cohort, assuming a dominant disease model. (**E**) Within the entire cohort, assuming an additive disease model. (**F**) Within individuals with predicted African heritage only, assuming a dominant disease model. (**G**) Within individuals with predicted African heritage only, assuming an additive disease model.

To investigate the biological significance of the SUZ12P1–CRLF3 variant, we assessed differences between genotyped groups using both *in vitro* and *in silico* data. In the GTEx population, we observed no significant difference in the expression levels of SUZ12P1 or CRLF3 (Figure 6A and B). Additionally, there was no clear distinction in exon usage, indicating no apparent difference in splicing isoforms between SUZ12P1–CRLF3+ and SUZ12P1–CRLF3− groups within an African American background (Supplementary Figure S3). Furthermore, no significant difference in the expression of other genes within this locus was identified (Supplementary Figure S4).

Expanding our analysis to physical characteristics, we examined individuals in the GTEx cohort and found no differences in height, weight or body mass index between those harboring the rearrangement allele and those without (Supplementary Figure S5A–C). Similarly, no differences were observed when making the same comparisons within the University of Virginia clinical donor cohort (Supplementary Figure S5D–F).

#### Association of SUZ12P1–CRLF3 allele with patient clinical codes

The human leukocyte samples we used have 234 unique clinical codes that describe historical health issues that each patient has exhibited. Among the leukocyte samples tested for the *SUZ12P1*–*CRLF3* chimeric RNA, 123 samples from African American individuals had linked clinical codes, including 14 of 15 samples testing positive for the *SUZ12P1*–*CRLF3* chimeric RNA. Although genetic data for these donors were unavailable, and assessment is limited by sample size, we assessed the difference in code occurrence between *SUZ12P1*–*CRLF3+*and *SUZ12P1*–*CRLF3−* donors to explore any potential links between *SUZ12P1*–*CRLF3* expression and clinical diagnoses. Controlling for gender, genotype-corrected inferred ancestry and age, we discovered a strong association (*P* = 0.038, OR = 12.1) of the rearrangement allele with ACD (Figure 6C).

#### SUZ12P1–CRLF3 and neurofibromatosis

Notably, the 17q11.2 locus is associated with NF1, caused by alterations within or microdeletions that span the *NF1* gene. Vasculopathy is a known indication of NF1, and cerebrovascular (43–46) manifestations have been associated with the disease. The three most common deletions occur between repetitive regions surrounding the *NF1* gene (Supplementary Figure S6A). In contrast, type II deletions occur via non-allelic homologous recombination between regions of the *SUZ12* polycomb gene and the *SUZ12P1* pseudogene. Due to scarcity of data characterizing type II NF1 deletions, we searched for a panel of SNPs associated with the variant to use as a proxy for the genotype. Significantly correlated SNPs clustered around the immediate locus at 17q11.2 (Supplementary Figure S7). No SNPs significantly associated with the rearrangement were associated with disease within ClinVar (47).

#### PheWAS in the All of Us Research Program

To further explore the relationship between the *SUZ12P1*–*CRLF3* variant and disease, we expanded our investigation to the All of Us Research Program, which has made commendable efforts to prioritize enrollment of racial and ethnic minorities, resulting in a highly diverse cohort (48). We utilized a subset of donors with srWGS, further limited to those with associated ancestry and structural variation predictions, as well as reported date of birth and sex at birth. To assess disease association of this genotype, we designed a PheWAS across the general ‘Disease’ condition, as well as the NF1 and ACD conditions, encompassing a total of 11 522 unique SNOMED codes, translated and collapsed to 1152 unique phecodes with PheMAP (37). The final cohort used in this study comprised up to 8108 individuals, including 37.4% with predicted African heritage, 4.4% individuals heterozygous for the rearrangement and <0.3% homozygous for the rearrangement (Supplementary Table S4). The median frequency of each phecode within the population was 13, with a median of 24 phecodes per donor (Supplementary Figure S8A and B).

We performed the PheWAS on the cohort in total, as well as within the ancestral background for each ancestry-specific SV, assuming both additive and disease models (Figure 6D–G). In each configuration, no phecodes were found to be associated with the *SUZ12P1*–*CRLF3* variant. This result did not replicate our earlier observation of the *SUZ12P1*–*CRLF3* variant with ACD in the clinical population.

Similar analyses were conducted on the tandem duplications that give rise to the *TFG*–*ADGRG7* (Supplementary Figure S9) and *TRPM4*–*PPFIA3* variants (Supplementary Figure S10). In these analyses as well, no association of either polymorphic variant with any phecodes included in the study was found within their respective ancestral populations, though an association of sideroblastic anemia was found with the *TRPM4*–*PPFIA3* variant in the full ancestral population, demonstrating the increased stringency of testing within the ancestral population alone. This result indicates that each of these variants, despite producing novel transcripts, may be functionally inert for these populations.

### Polymorphic chimeric RNAs as predictors of transcribed gene fusions

#### Population-filtered chimeric RNAs are robust indicators for rare SVs

**Figure 7.**
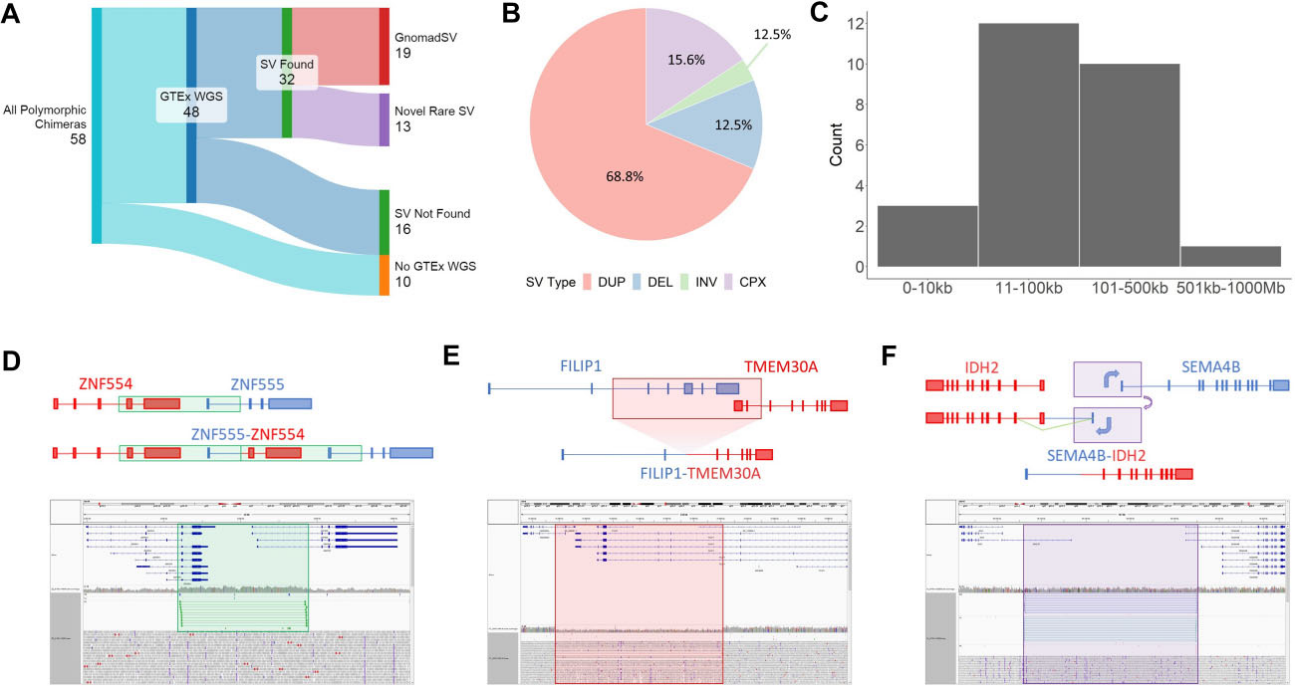
Population-filtered chimeric RNAs are robust indicators for rare SVs. (**A**) Sankey diagram summarizing overall findings of SV from WGS data. Created with SankeyMATIC (SankeyMATIC.com). (**B**) Pie chart indicating the frequency of SV types identified within validated SVs. (**C**) Histogram of validated SV size. (**D**) Cartoon depicting the *ZNF555*–*ZNF554* chimeric RNA, the SV that produces it and read alignment over the genomic locus in IGV. The duplicated region is highlighted in green. (**E**) Cartoon and read alignments depicting the *FILIP1*–*TMEM30A* and (**F**) *SEMA4B*–*IDH2* chimeric RNAs. Deleted regions are indicated in red and inverted regions are indicated in purple.

Based on our investigation into the origins of the SUZ12P1–CRLF3 chimeric transcript, we sought to determine the prevalence of other population-specific chimeric RNAs resulting from large-scale SVs. Among the 58 population-specific chimeric RNAs, 48 were found in GTEx donors with matching WGS data. Through analysis of discordant read pairs within each region of interest, we observed direct evidence of 29 SVs accounting for 32 (66.6%) of the predicted polymorphic chimeras with WGS data (Figure 7A and Supplementary Figure S11). Notably, we did not find evidence for chimeras predicting interchromosomal rearrangements, which are more likely to be spurious based on our experience. Among the 16 chimeras for which evidence of SVs was not found, 11 comprised interchromosomal gene pairs. Moreover, we were able to identify GnomadSV (49) annotations for 19 of 32 validated variants, including 18 rare variants (MAF < 1%) (Supplementary Table S2), leaving 13 rare variants that, to our knowledge, are novel.

We find that the majority of these variants are duplications, while a smaller proportion of SVs are deletions, inversions or complex combinations of these components (Figure 7B). Additionally, these SVs predominantly fell between 10 and 500 kb, with a few exceptions of small SVs (<10 kb) and one large SV (>500 kb) (Figure 7C). We highlight three additional polymorphic chimeric RNAs and the SVs that produce them.

1. *ZNF555*–*ZNF554* is produced by a tandem duplication, which creates an additional fusion gene in between the two parental genes on the subject genome (Figure 7D). This rearrangement is evidenced by a pile-up of right–left aligned reads at each breakpoint as well as increased read coverage within this region, indicating a copy number increase.
2. *FILIP1*–*TMEM30A* is generated by an interstitial deletion, which removes exons from the end of *FILIP1* as well as the beginning of *TMEM30A*, resulting in a singular fusion gene on the locus (Figure 7E).
3. *SEMA4B*–*IDH2* is created by an interstitial inversion, which relocates the promoter region and first exon from some *SEMA4B* isoforms upstream and onto the same strand of the *IDH2* gene, resulting in a chimeric transcript (Figure 7F).

Alongside these three examples, we provide direct evidence of SVs for an additional 26 chimeric RNAs (Figure 4B and Supplementary Figure S11), demonstrating a diverse range of mechanisms for fusion gene generation. For instance, *SLC25A33*–*TMEM201* and *SHPK*–*TRPV1* appear to harbor transcription termination site-specific deletions, and *CHD5*–*KCNAB2*/*KCNAB2*–*CHD5* result from a complex duplication and inversion, creating reciprocal fusion genes.

## Discussion

Chimeric RNAs have long been recognized as crucial players in various diseases, especially cancer. However, recent efforts have also explored their presence in healthy individuals, aiming to create a comprehensive resource for chimeric RNA characterization in non-diseased donors, which remains a relatively unexplored territory (50). In our study, we leveraged a healthy subgroup to identify 58 population-specific chimeric RNAs, including 2 novel African ancestral chimeras, and 13 novel SVs producing transcribed polymorphic chimeric RNAs. Notably, we conducted an in-depth analysis of *SUZ12P1*–*CRLF3*, shedding light on the rearrangement responsible for generating the chimeric RNA, and its associations in large global, regional and clinical cohorts.

An intriguing observation from our study was the identification of three donors who tested positive for the chimeric RNA but did not harbor the variant genotype. This suggests the possibility of the transcript being created via intergenic splicing, alongside the canonical transcription from a fusion gene. This notion is supported by the increased expression in samples with the permanent, transformed variant. This places *SUZ12P1*–*CRLF3* within a select group of chimeric RNAs, including *JAZF1*–*JJAZ1* (3), *PAX3*–*FOXO1* (5) and *EWSR1*–*FLI1* (51). These chimeras form the basis for the ‘cart before the horse’ hypothesis (52), wherein a pre-existing chimeric transcript could mediate genomic translocation events. Growing evidence supports this idea, including controlled repair of a GFP transgene (53), and direct evidence of chimeric RNA-mediated generation of the canonical *TMPRSS2*–*ERG* and *TMPRSS2*–*ETV1* gene fusions (54).

While we found no relationship between the *SUZ12P1*–*CRLF3* genomic variant with NF1, our search for associated SNPs provided a fortuitous finding in Rs145766379_A_G, which is located within the downstream breakpoint region of the inversion. Interestingly, we found that Rs145766379_A_G introduces a region of increased likelihood for secondary structure formation near the observed breakpoint. Paired with other observations of the region, including sequence homology between Alu repeat elements, we have formed a working theory for the origination of the rearrangement (Supplementary Figure S6B–F). However, further testing is required to confirm or reject the hypothesis.

Our method provides a targeted, bottom-up means for identifying gene fusion transcripts that can be analyzed similarly. This stands in contrast to top-down approaches focusing on variants that overlap gene annotations, which cannot be assumed to produce a fusion transcript. Such top-down approaches may face limitations due to reference biases or alignment blackout regions. We believe our methodology will be valuable for similar analyses in other datasets.

This report provides a framework for further characterization studies, both for chimeric RNAs not fully explored here and for other model systems and cohorts. The 58 population-specific chimeric RNAs identified in this study likely represent only a fraction of observable events that can be uncovered with bottom-up approaches. Expanding this catalog could be achieved by incorporating additional chimeric RNA prediction algorithms. Moreover, advancements in comprehensive SV categorization allow for automation into the filtering pipeline. Thus, this analytical approach complements top-down SV prediction, particularly when targeting potentially functional variants producing fusion genes.

## Supplementary Material

gkae258_Supplemental_Files

## Data Availability

No new data were generated in support of this research. Scripts related to this project have been deposited in Figshare at https://figshare.com/projects/PolymorphicChimera/193688.
