# Supplementary material for: Discovery of a polymorphic gene fusion via bottom-up chimeric RNA prediction
Source: Nucleic Acids Res. 2024 Apr 8;52(8):4409–21. doi: 10.1093/nar/gkae258 (PMC11077074; doi:10.1093/nar/gkae258)
Supplement: gkae258_Supplemental_Files [file gkae258_supplemental_files.zip › SupplementalFigures_revised_final_Apr04.pdf]

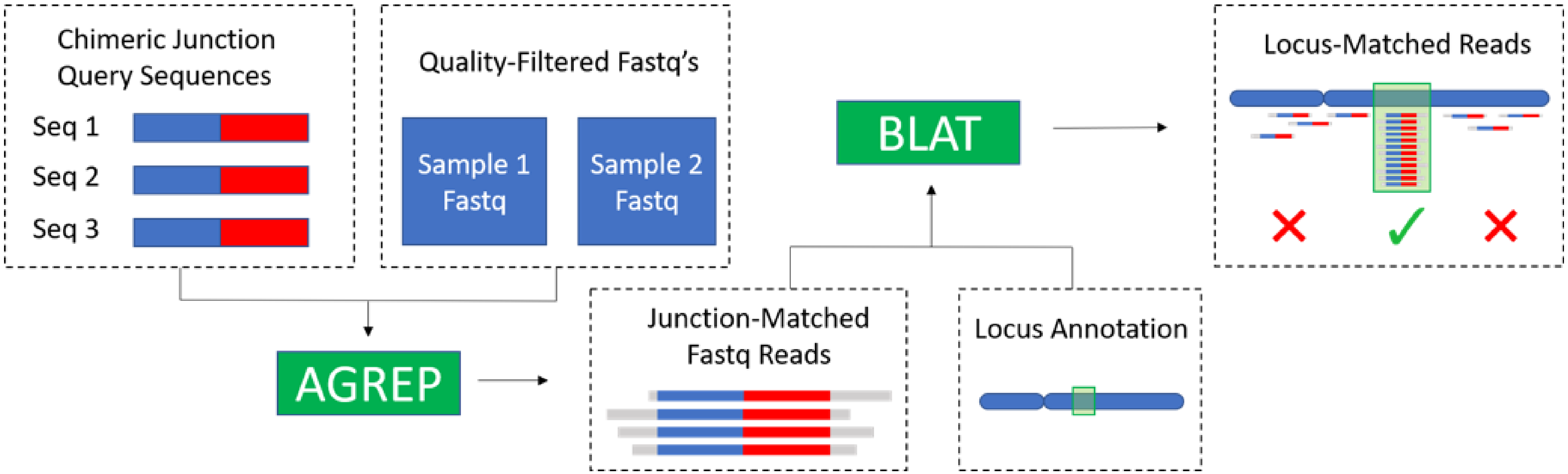

Chimeric Junction
Query Sequences
Seq 1
Seq 2
Seq 3
Quality-Filtered Fastq's
Sample 1
Fastq
Sample 2
Fastq
BLAT
Locus-Matched Reads
AGREP
Junction-Matched
Fastq Reads
Locus Annotation

SNP Agreement		Sample Designation	
Yes	*No*	*Unchanged*	*Changed*
15	*0*	*9*	*6*

Changes in Sample Designation					
$W \rightarrow N$	$W \rightarrow U$	$B \rightarrow E$	$B \rightarrow U$	$U \rightarrow E$	$U \rightarrow N$
1	*0*	*0*	*0*	*0*	*5*

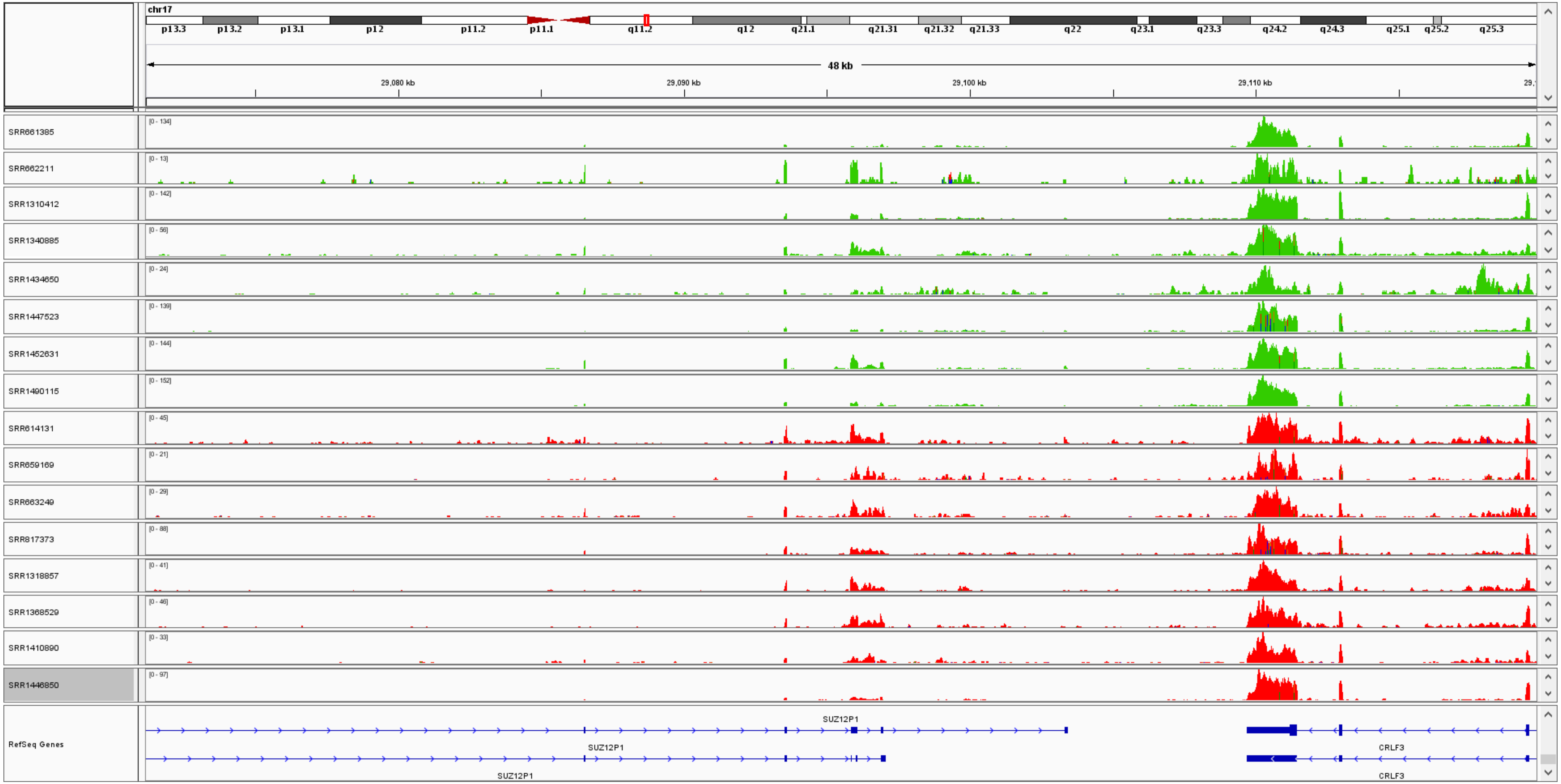
chr17
p13.3
p13.2
p13.1
p12
p11.2
p11.1
q11.2
q12
q21.1
q21.31
q21.32
q21.33
q22
q23.1
q23.3
q24.2
q24.3
q25.1
q25.2
q25.3
48 kb
29,080 kb
29,090 kb
29,100 kb
29,110 kb
SRR661385
[0 - 134]
SRR662211
[0 - 13]
SRR1310412
[0 - 142]
SRR1340885
[0 - 56]
SRR1434650
[0 - 24]
SRR1447523
[0 - 139]
SRR1452631
[0 - 144]
SRR1490115
[0 - 152]
SRR614131
[0 - 45]
SRR659169
[0 - 21]
SRR663249
[0 - 29]
SRR817373
[0 - 88]
SRR1318857
[0 - 41]
SRR1368529
[0 - 46]
SRR1410890
[0 - 33]
SRR1446850
[0 - 97]
RefSeq Genes
SUZ12P1
SUZ12P1
SUZ12P1
CRLF3
CRLF3

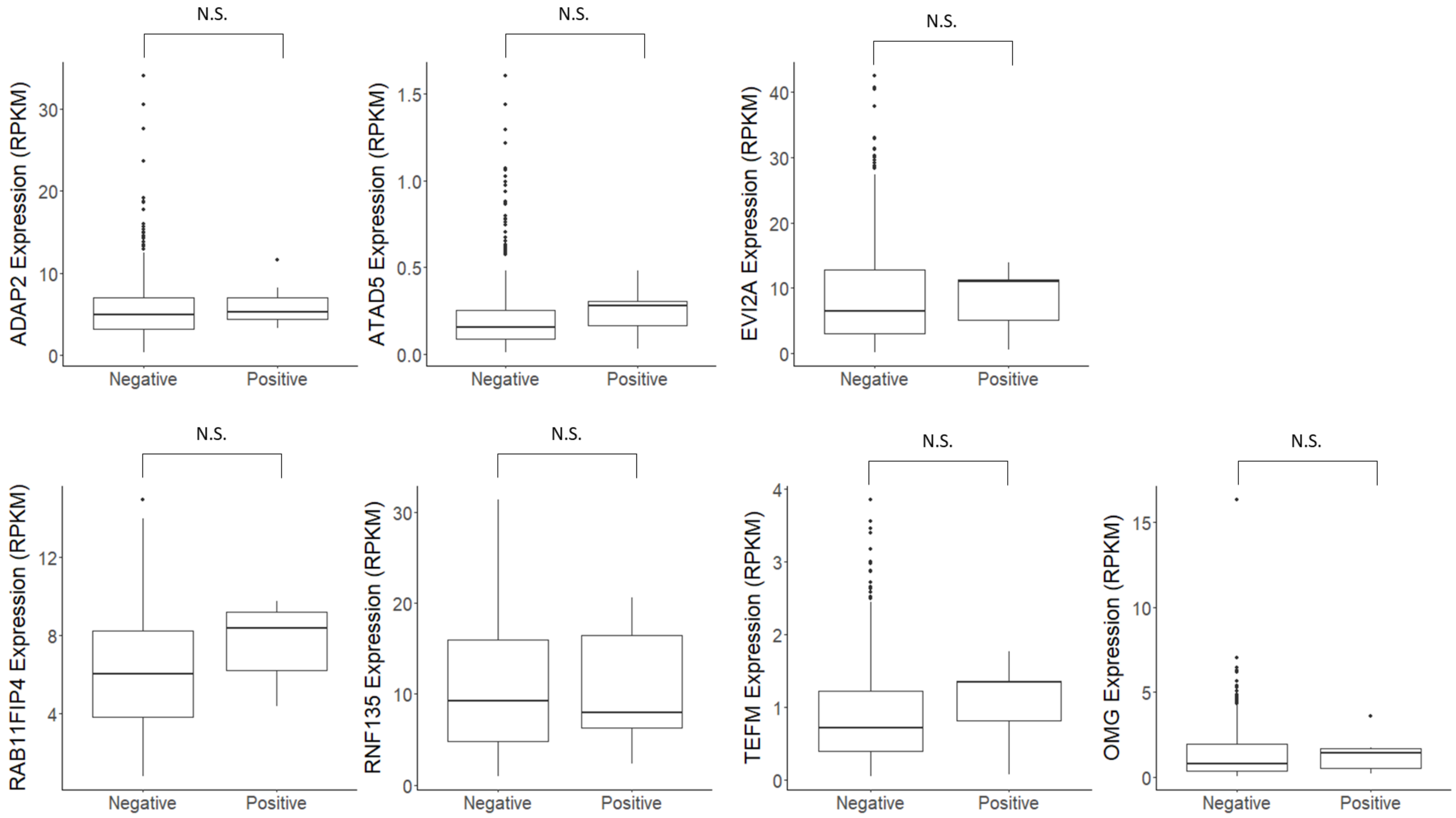

N.S.
ADAP2 Expression (RPKM)
0
10
20
30
Negative
Positive
N.S.
ATAD5 Expression (RPKM)
0.0
0.5
1.0
1.5
Negative
Positive
N.S.
EVI2A Expression (RPKM)
0
10
20
30
40
Negative
Positive
N.S.
RAB11FIP4 Expression (RPKM)
4
8
12
Negative
Positive
N.S.
RNF135 Expression (RPKM)
0
10
20
30
Negative
Positive
N.S.
TEFM Expression (RPKM)
0
1
2
3
4
Negative
Positive
N.S.
OMG Expression (RPKM)
0
5
10
15
Negative
Positive

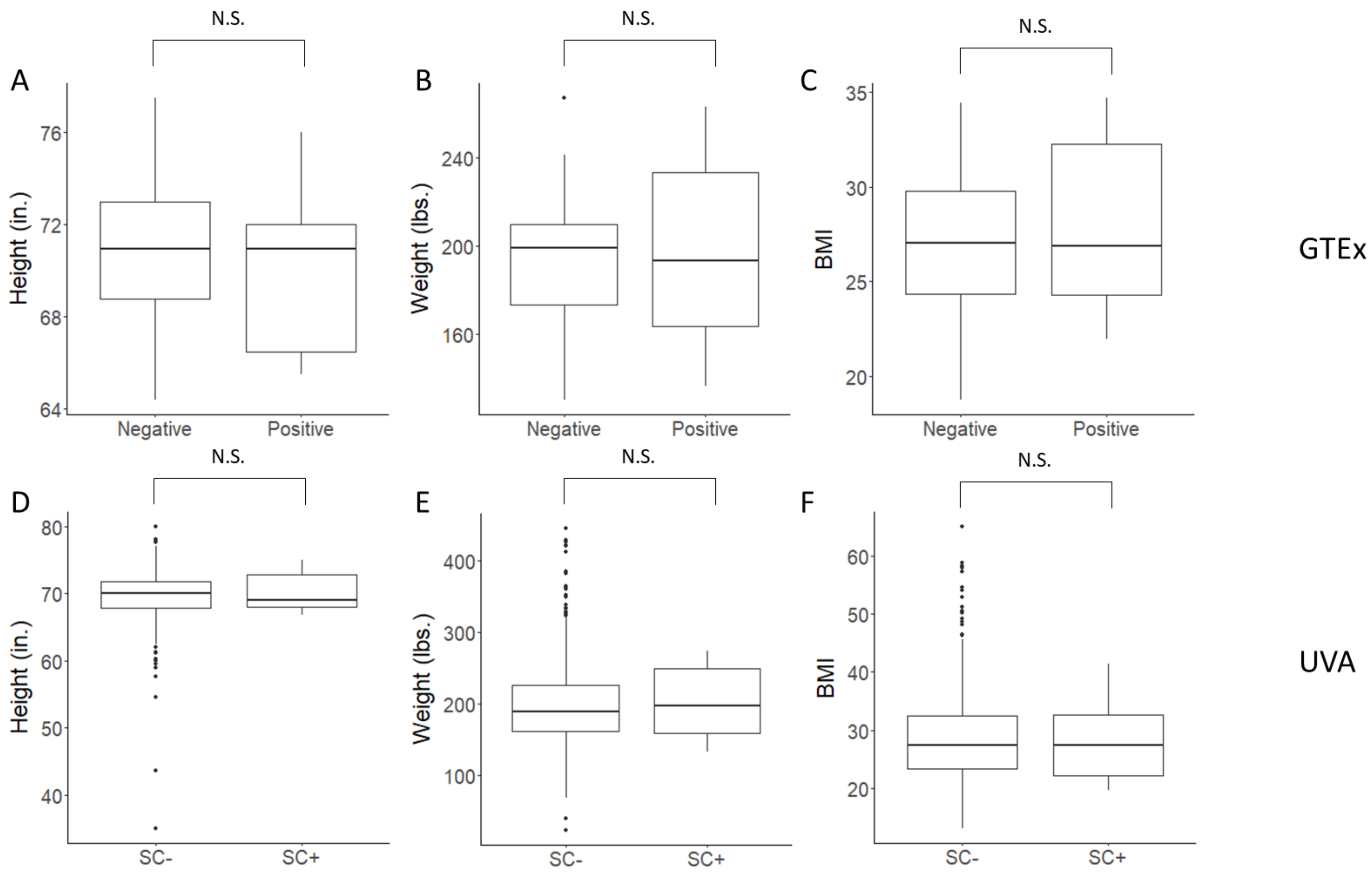

N.S.
A
Height (in.)
76
72
68
64
Negative
Positive
N.S.
B
Weight (lbs.)
240
200
160
Negative
Positive
N.S.
C
BMI
35
30
25
20
Negative
Positive
GTEx
N.S.
D
Height (in.)
80
70
60
50
40
SC-
SC+
N.S.
E
Weight (lbs.)
400
300
200
100
SC-
SC+
N.S.
F
BMI
60
50
40
30
20
SC-
SC+
UVA

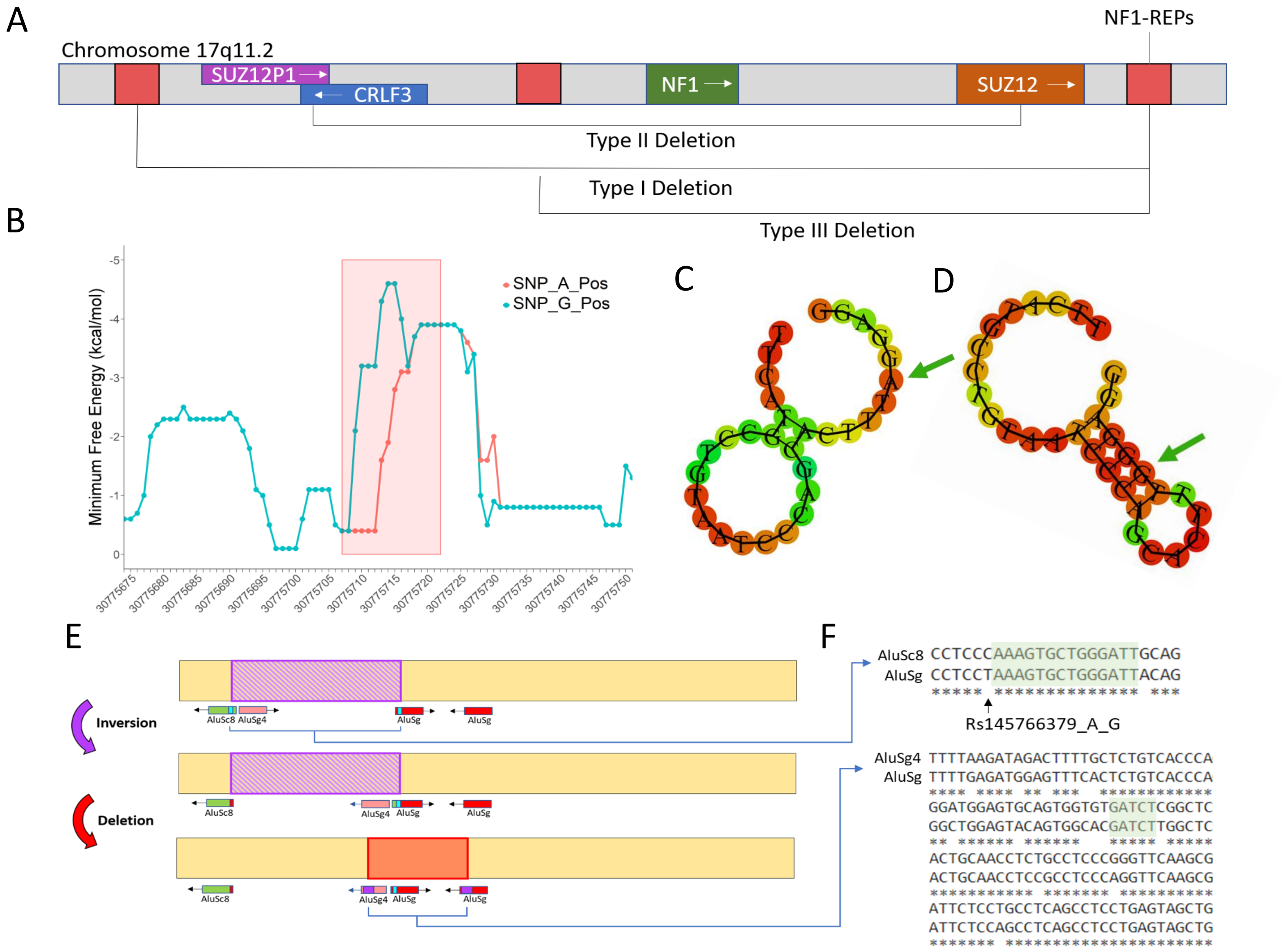

A
NF1-REPs
Chromosome 17q11.2
SUZ12P1
CRLF3
NF1
SUZ12
Type II Deletion
Type I Deletion
Type III Deletion
B
Minimum Free Energy (kcal/mol)
SNP_A_Pos
SNP_G_Pos
30775675
30775680
30775685
30775690
30775695
30775700
30775705
30775710
30775715
30775720
30775725
30775730
30775735
30775740
30775745
30775750
C
D
E
Inversion
Deletion
AluSc8
AluSg4
AluSg
F
AluSc8 CCTCCCAAAGTGCTGGGATTGCAG
AluSg CCTCCTAAAGTGCTGGGATTACAG
Rs145766379_A_G
AluSg4 TTTTAAGATAGACTTTTGCTCTGTCACCCA
AluSg TTTTGAGATGGAGTTTCACTCTGTCACCCA
GGATGGAGTGCAGTGGTGTGATCTCGGCTC
GGCTGGAGTACAGTGGCACGATCTTGGCTC
ACTGCAACCTCTGCCTCCCGGGTTCAAGCG
ACTGCAACCTCCGCCTCCCAGGTTCAAGCG
ATTCTCCTGCCTCAGCCTCCTGAGTAGCTG
ATTCTCCAGCCTCAGCCTCCTGAGTAGCTG

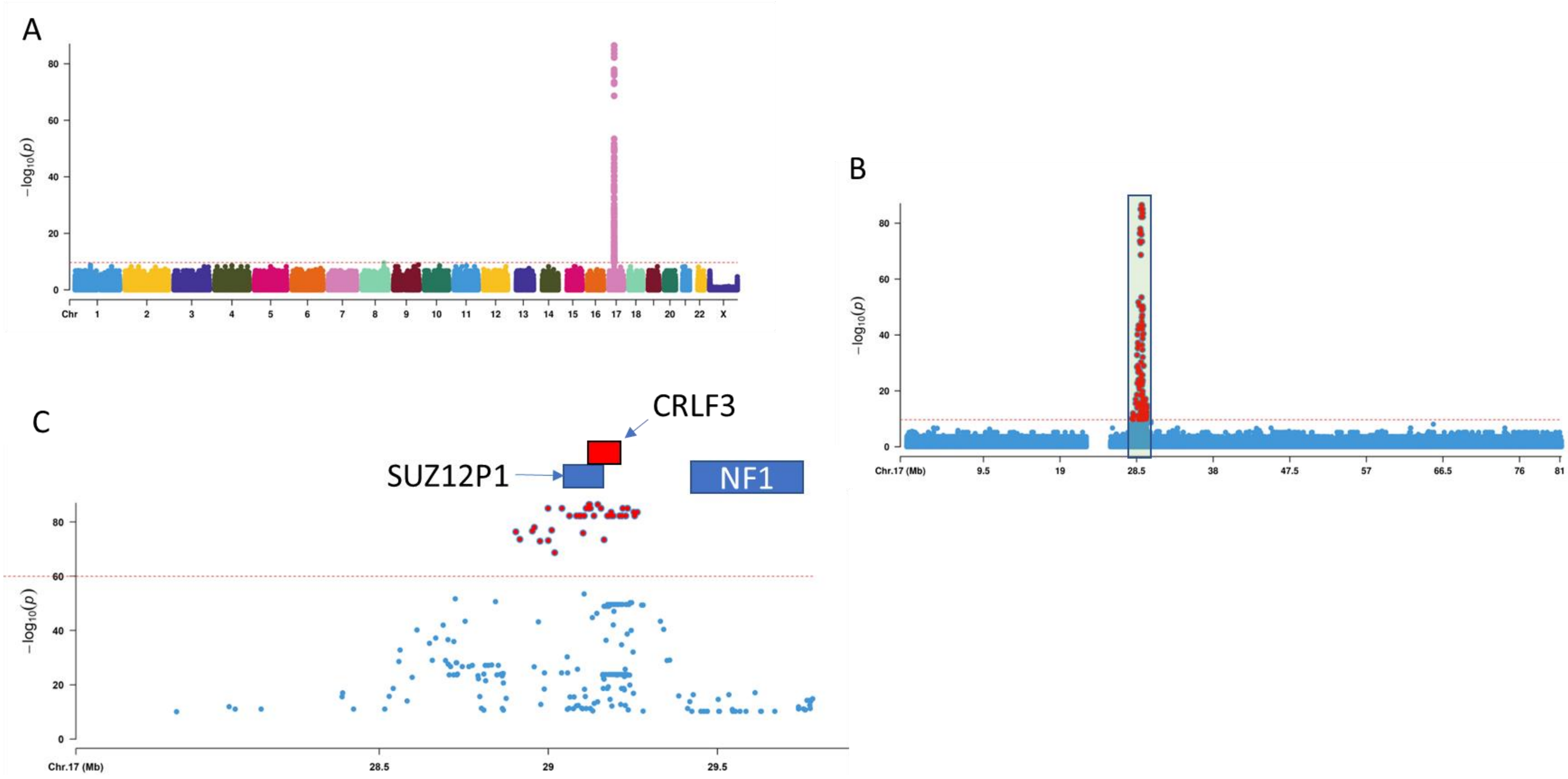
A
−log10(p)
80
60
40
20
0
Chr 1 2 3 4 5 6 7 8 9 10 11 12 13 14 15 16 17 18 20 22 X
B
−log10(p)
80
60
40
20
0
Chr.17 (Mb) 9.5 19 28.5 38 47.5 57 66.5 76 81
C
CRLF3
SUZ12P1
NF1
−log10(p)
80
60
40
20
0
Chr.17 (Mb) 28.5 29 29.5

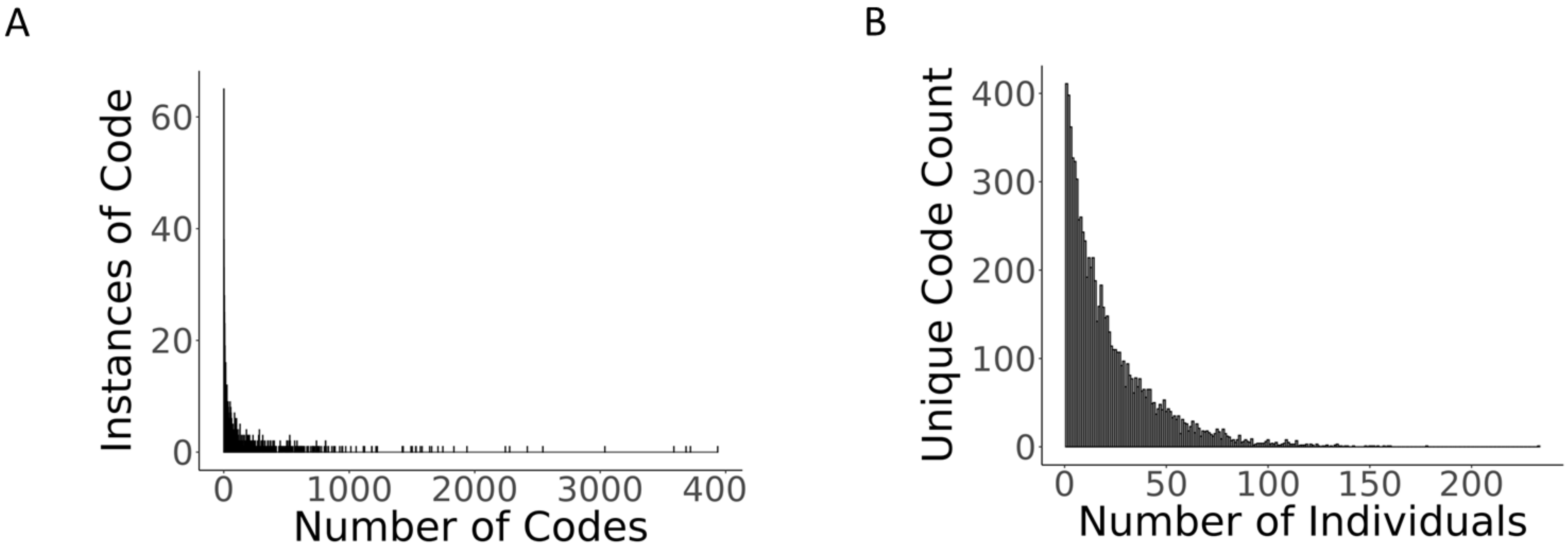
A
60
40
20
0
Instances of Code
0
1000
2000
3000
400
Number of Codes
B
400
300
200
100
0
Unique Code Count
0
50
100
150
200
Number of Individuals

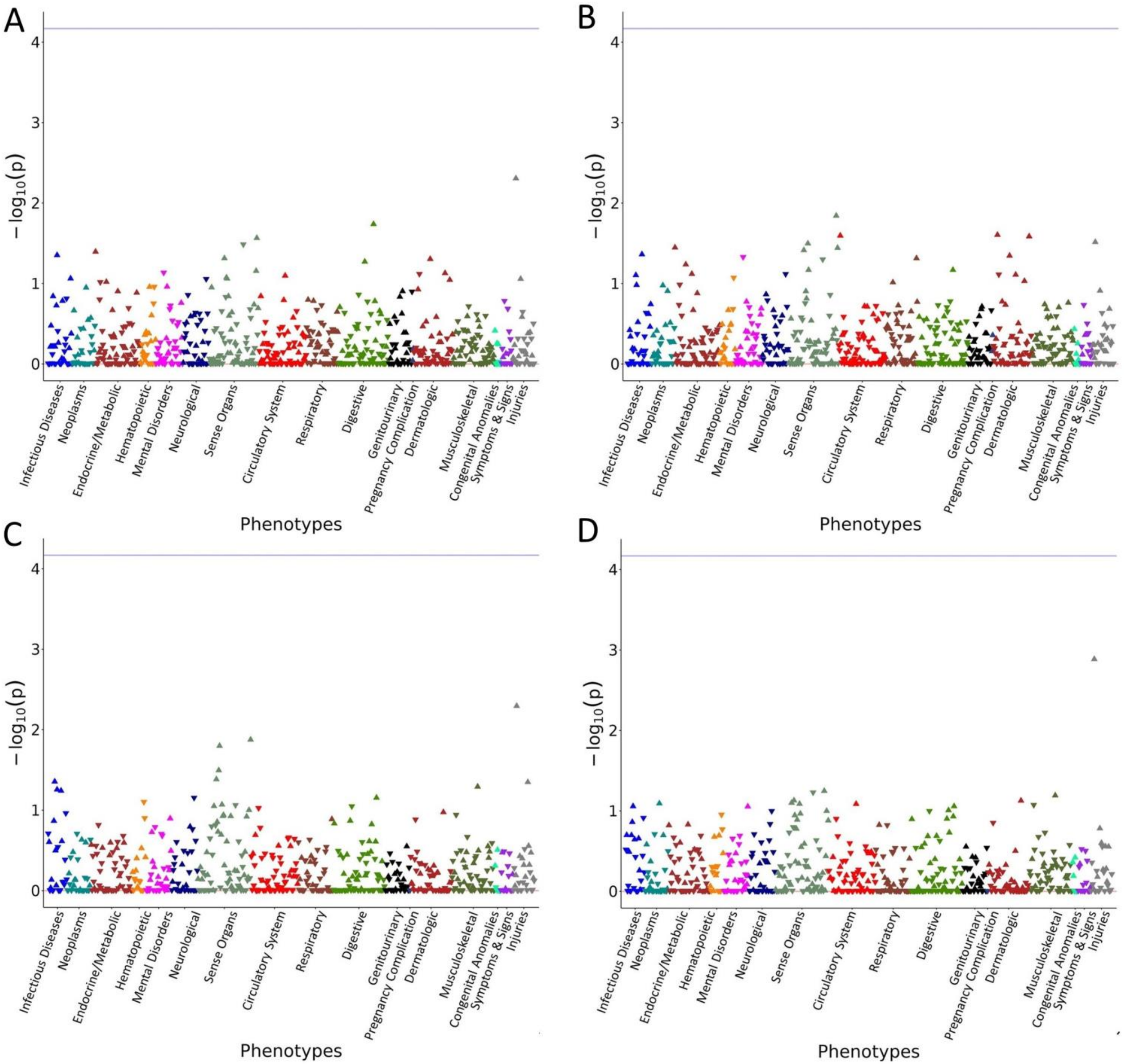
A
B
C
D
−log10(p)
Phenotypes
Infectious Diseases
Neoplasms
Endocrine/Metabolic
Hematopoietic
Mental Disorders
Neurological
Sense Organs
Circulatory System
Respiratory
Digestive
Genitourinary
Pregnancy Complication
Dermatologic
Musculoskeletal
Congenital Anomalies
Symptoms & Signs
Injuries

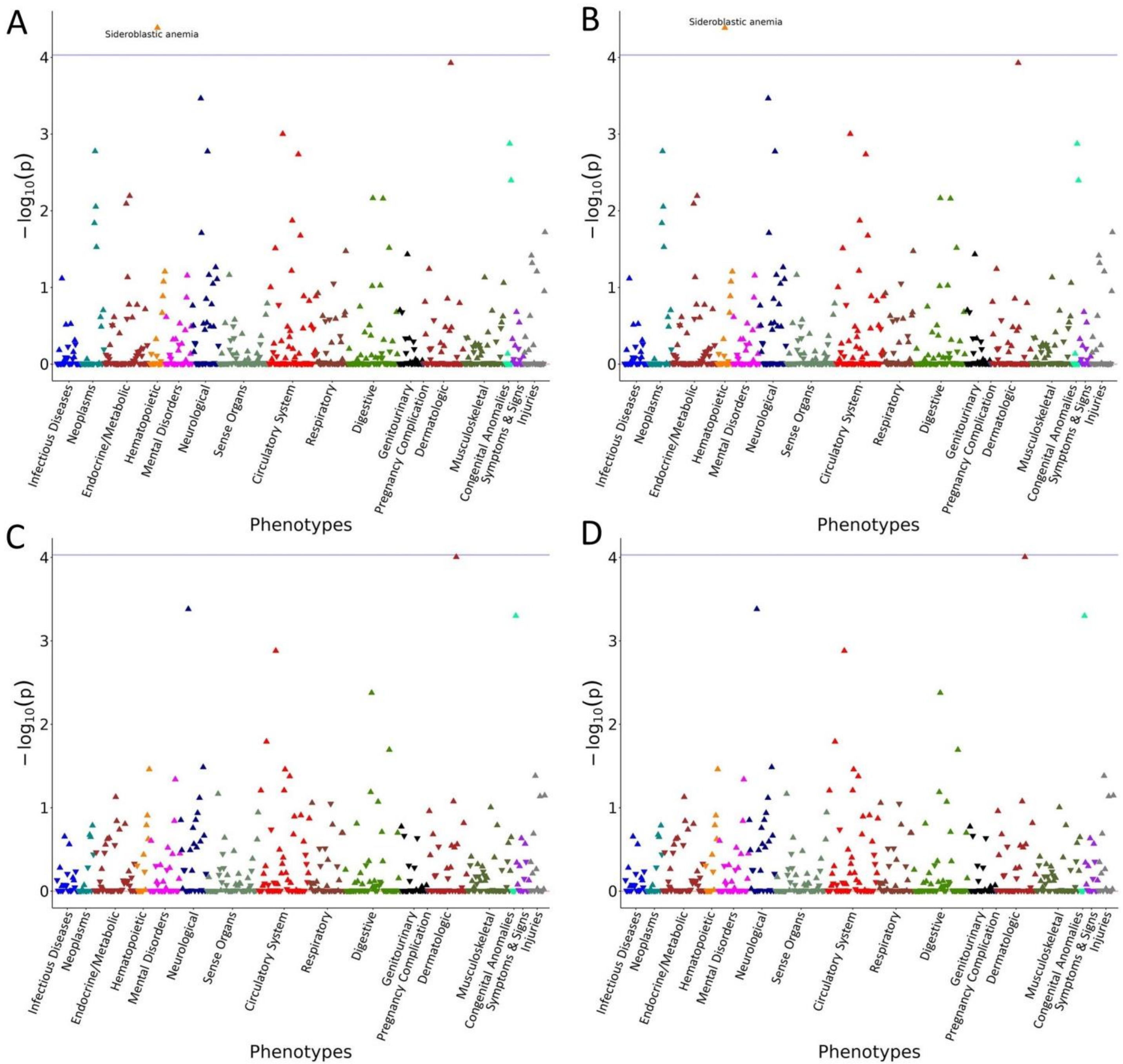
A
B
C
D
Sideroblastic anemia
Sideroblastic anemia
$-\log_{10}(p)$
Phenotypes
Infectious Diseases
Neoplasms
Endocrine/Metabolic
Hematopoietic
Mental Disorders
Neurological
Sense Organs
Circulatory System
Respiratory
Digestive
Genitourinary
Pregnancy Complication
Dermatologic
Musculoskeletal
Congenital Anomalies
Symptoms & Signs
Injuries

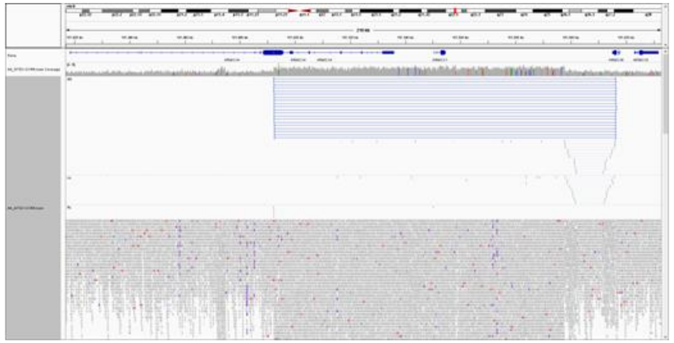

ARMCX6-ARMCX4

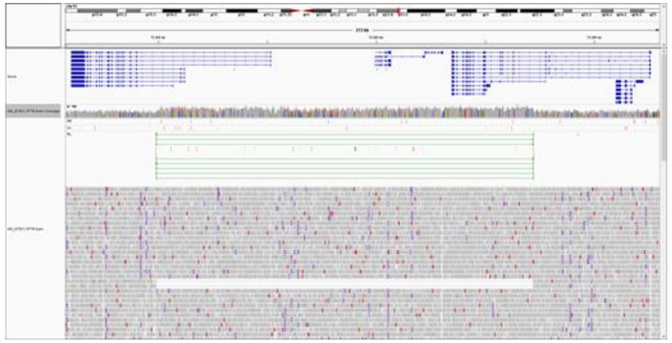

ARRB1-GDPD5

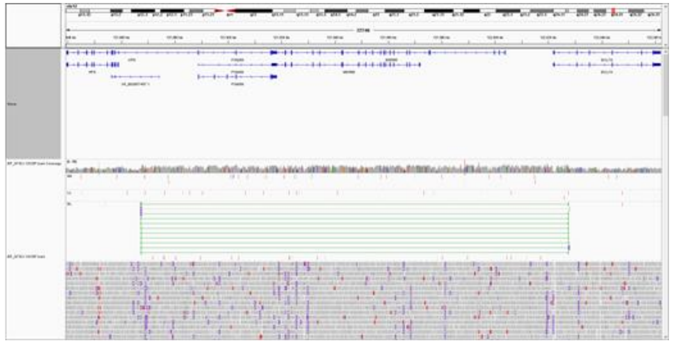

BCL7A-PSMD9

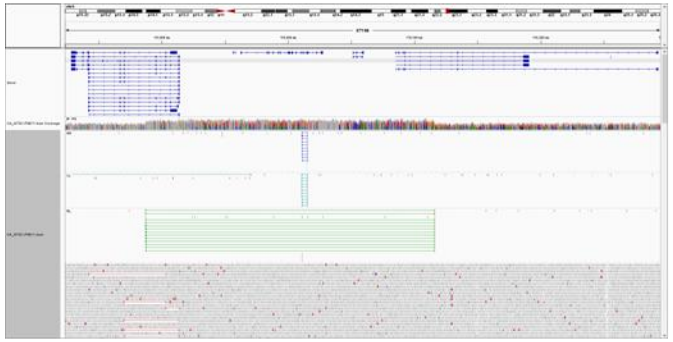

COMMD10-AP3S1

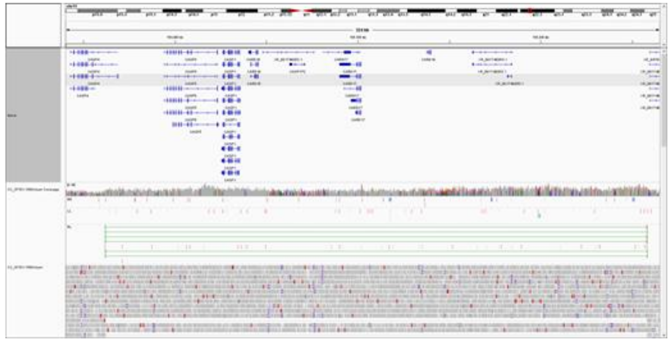

CASP4-CARD18

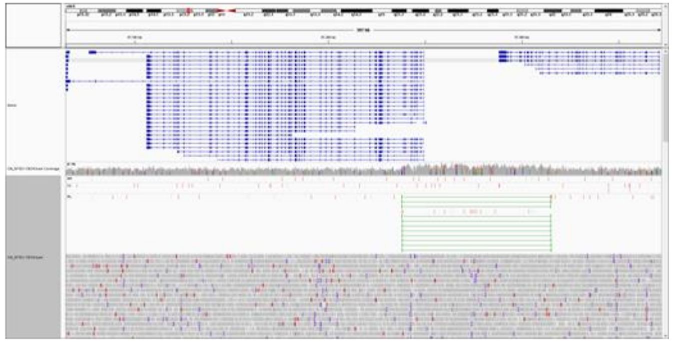

C5orf42 (CPLANE1)-NUP155

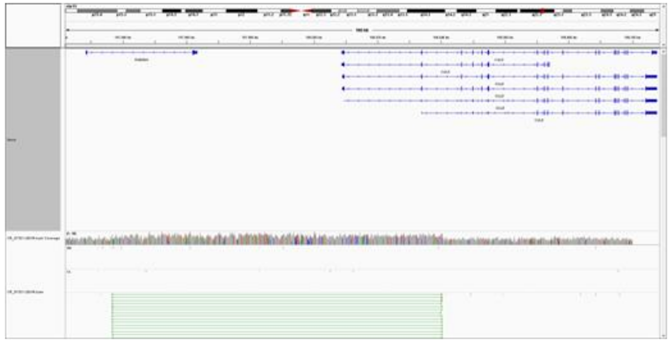

CUL5-RAB39A

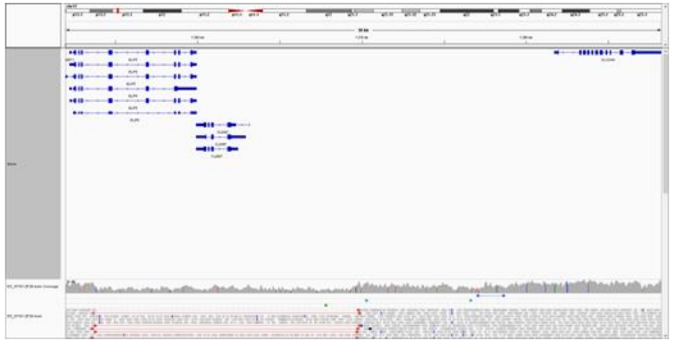

ELP5-SLC2A4

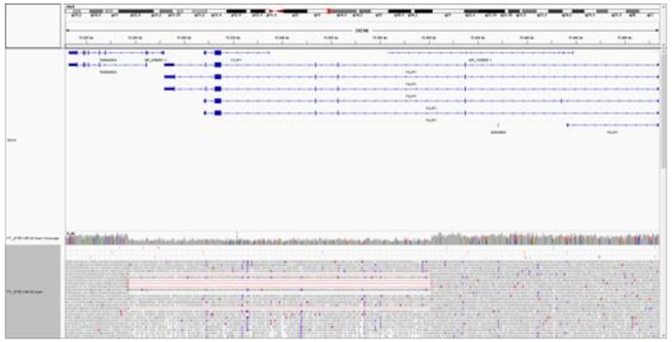

FILIP1-TMEM30A

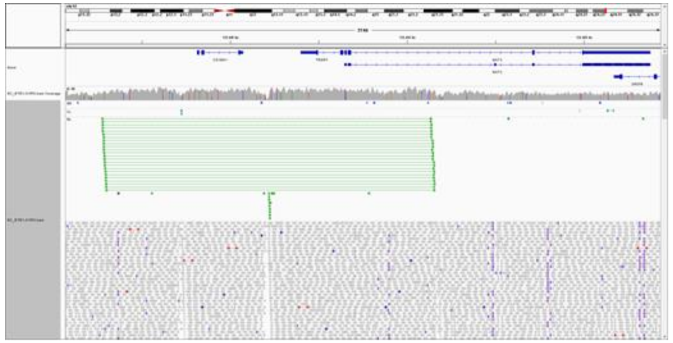

GATC-COX6A1

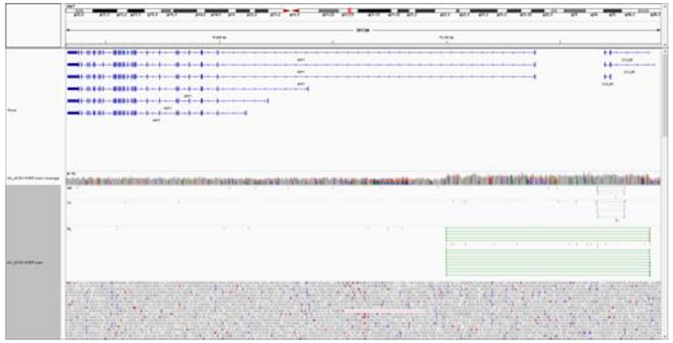

HIP1-CCL26

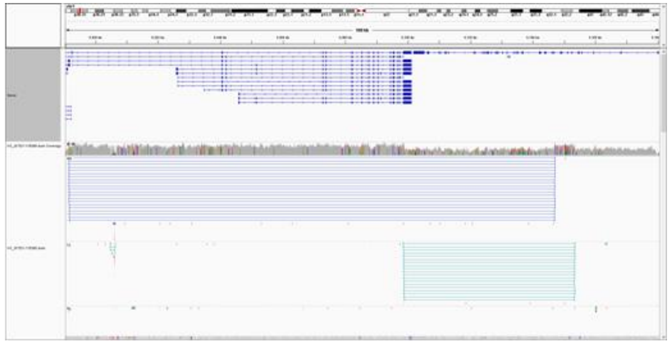

CHD5-KCNAB2
KCNAB2-CHD5

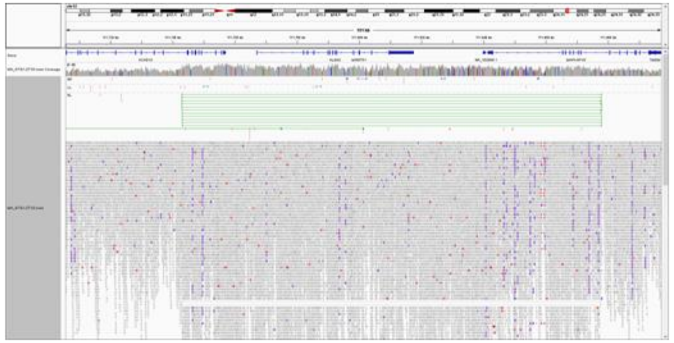

MAPKAPK5-ACAD10

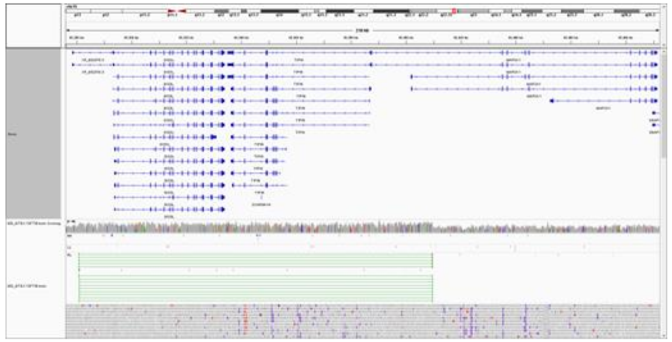

MAP2K1-DIS3L

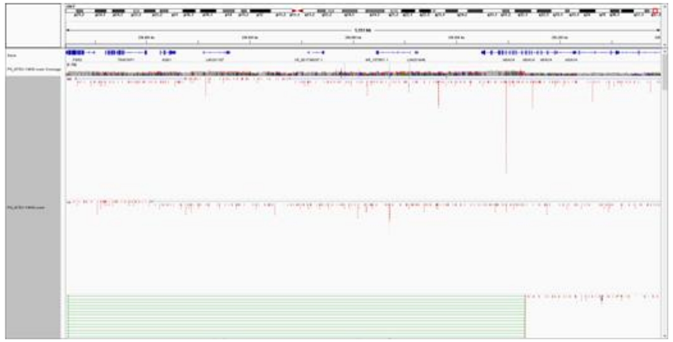

PER2-HDAC4

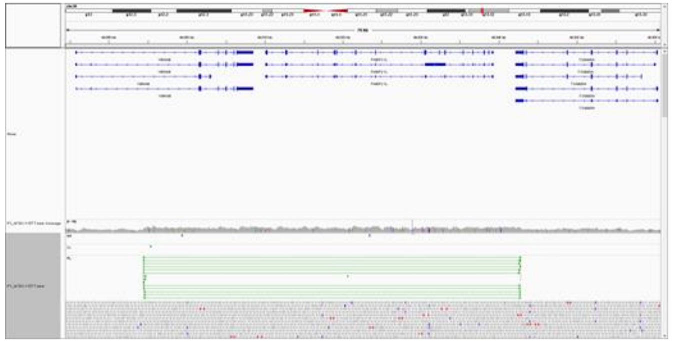

PABPC1L-YWHAB

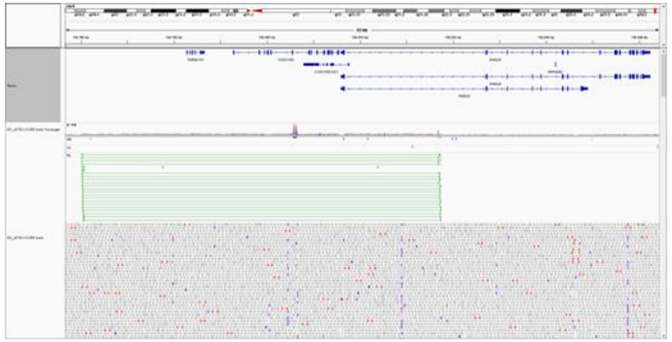

RABL6-CCDC183
RABL6-TMEM141

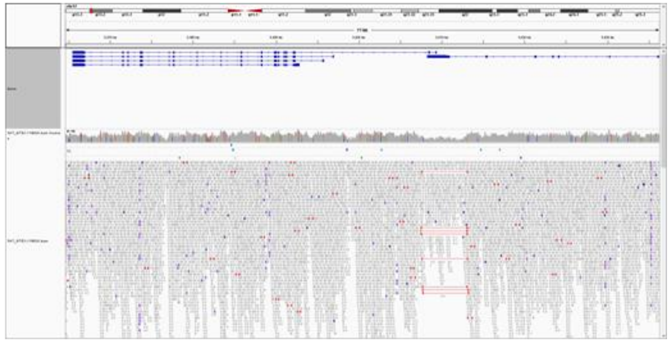

SHPK-TRPV1

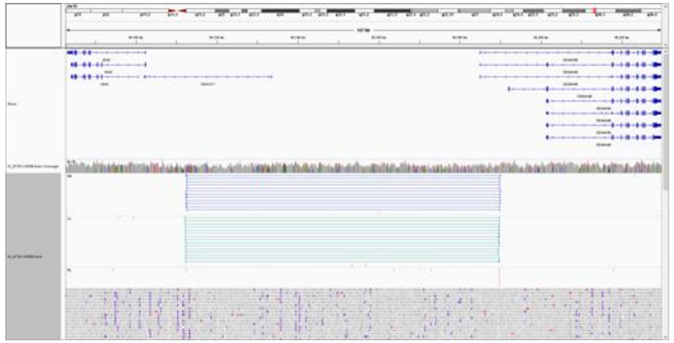

SEMA4B-IDH2

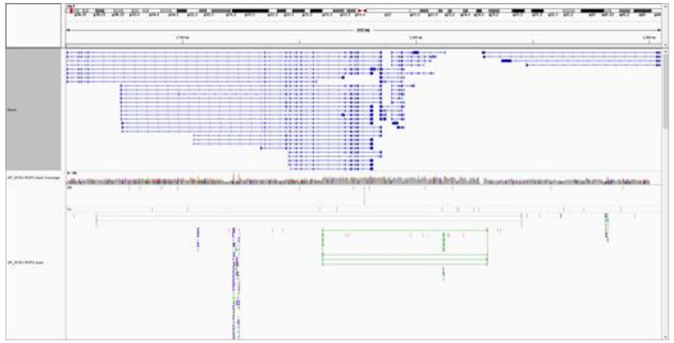

SKI-PRKCZ

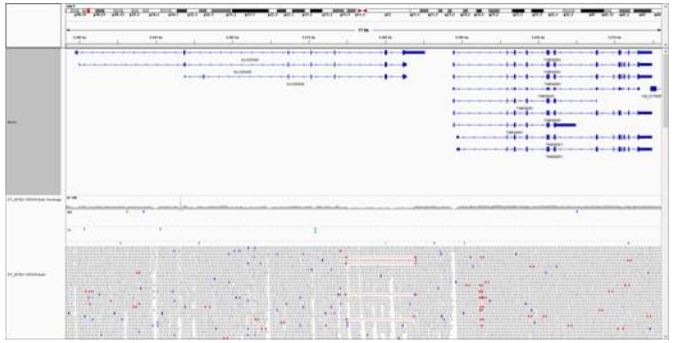

SLC25A33-TMEM201

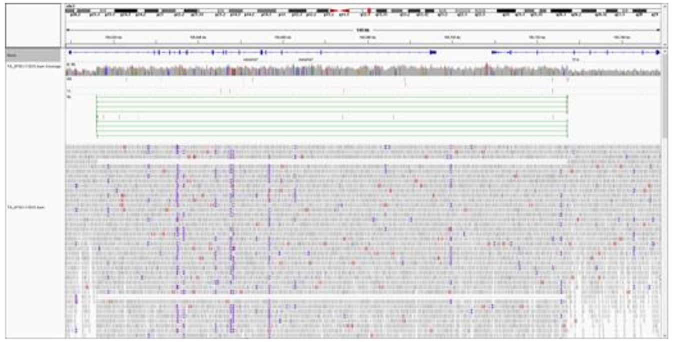

TFG-ADGRG7

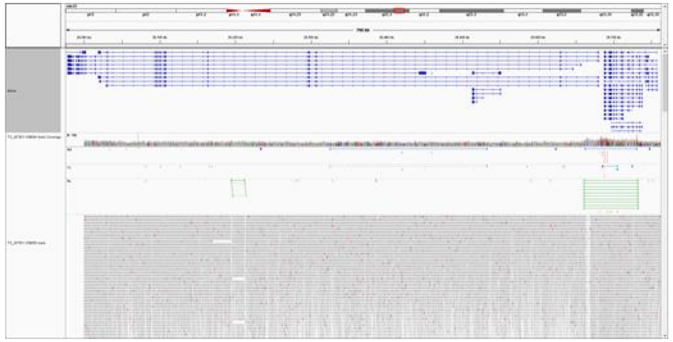

TTC28-CHEK2

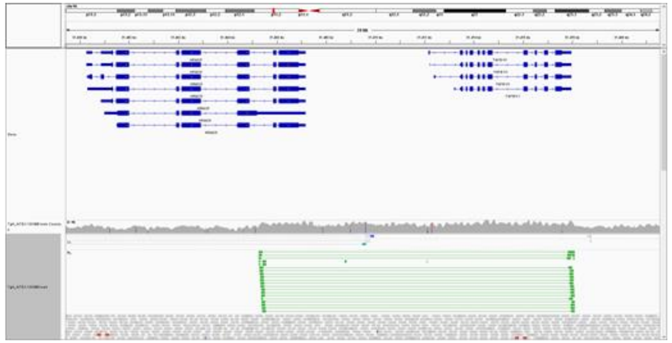

TGFB1I1-ARMC5

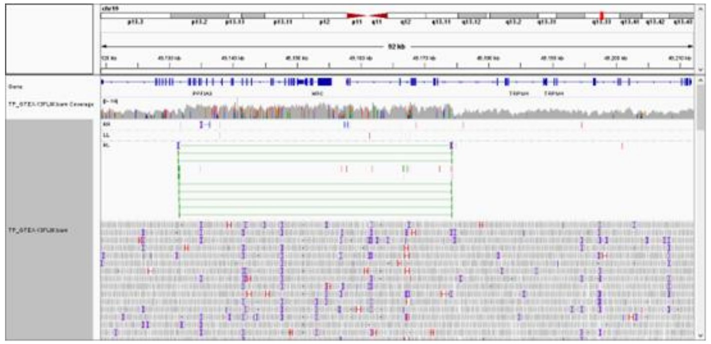

TRPM4-PPFIA3

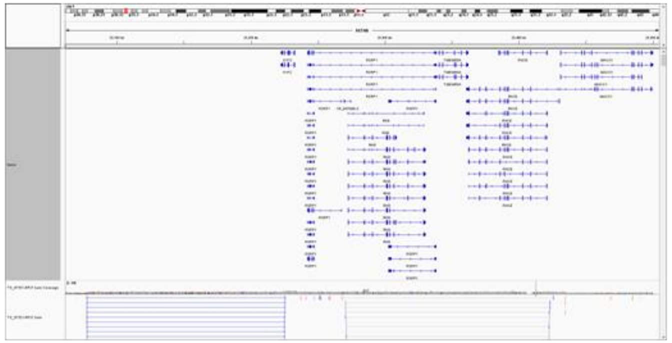

TMEM57 (MACO1)-SYF2

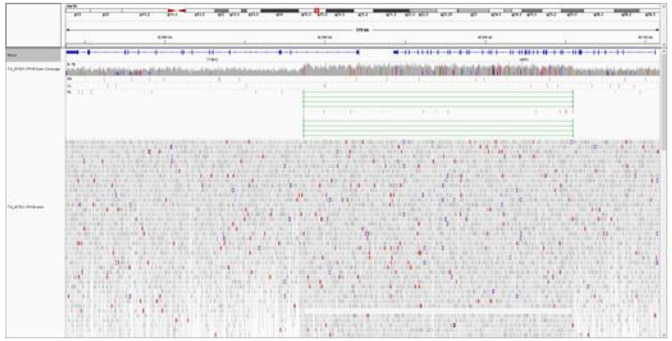

TTBK2-UBR1

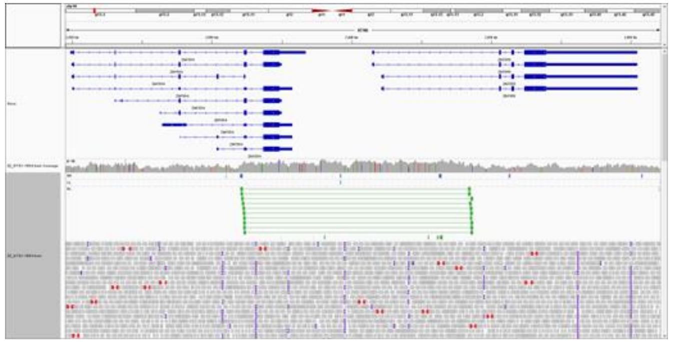

ZNF555-ZNF554
