# Supplementary material for: Discovery of a polymorphic gene fusion via bottom-up chimeric RNA prediction
Source: Nucleic Acids Res. 2024 Apr 8;52(8):4409–21. doi: 10.1093/nar/gkae258 (PMC11077074; doi:10.1093/nar/gkae258)
Supplement: gkae258_Supplemental_Files [file gkae258_supplemental_files.zip › SupplementaryTableAndFigureLegends_25MAR2024_Clean.docx]

**SUPPLEMENTARY TABLE AND FIGURES LEGENDS**

Supplementary Table S1. List of Primers Used.

Supplementary Table S2. Population-specific chimeric RNA predictions and SV annotation.

Supplementary Table S3. Genotype and allele frequency in 1000 genomes populations with *SUZ12P1-CRLF3*-expressing individuals.

Supplementary Table S4. All of Us donor cohort characteristics, masking crosstabulations with fewer than 20 individuals per All of Us statistical dissemination policy.

Supplementary Table S5. AGREP query sequences used for *in silico* genotyping of *SUZ12P1-CRLF3*, *TFG-ADGRG7*, and *TRPM4-PPFIA3* variants.

Supplementary Figure S1. AGREP pipeline diagram.

Supplementary Figure 2. Changes to sample designation resulting from SNP genotying. W = White, B = Black or African American, U = Unknown, N = Non-European, E = European.

Supplementary Figure S3. *SUZ12P1* and *CRLF3* read coverage by SC genotype. Individuals found to possess the SUZ12P1-CRLF3 variant are represented in green, and those who do not possess the SUZ12P1-CRLF3 variant are represented in red.

Supplementary Figure S4. Gene expression of genes on the 17q11.2 locus by SC genotype.

Supplementary Figure S5. Donor characteristics in GTEx and UVA cohorts stratified by SC genotype. GTEx donor A) height, B) weight, and C) BMI by SC genotype. UVA donor D) height, E) weight, and F) BMI by SC genotype.

Supplementary Figure S6. The NF1 genomic locus and theorized origination for the *SUZ12P1-CRLF3 variant.* A) Annotation of genes and repetitive elements on 17q11.2 relevant to microdeletions in type I neurofibromatosis. B) RNAfold secondary structure prediction minimum free energy (MFE) calculations for the locus. Predictions are made using a 30 bp sliding window at each base pair surrounding Rs145766379_A_G and are calculated using reference (A, red) and variant (G,blue) alleles. The window of homology depicted in panel J is highlighted in red. C) RNAfold models of the 30 bp window including Rs145766379_A and D) Rs145766379_G. E) The SUZ12P1-CRLF3 locus with annotated Alu elements. The rearrangement was likely caused by an inversion followed by a deletion mediated by F) homologous sequence at each breakpoint, shown in light blue for the inversion and purple for the deletion. Regions containing the possible exact breakpoints are highlighted in green.

Supplementary Figure S7. Manhattan plots of SNPs associated with the SC variant. A) Displaying variants across HG38, B) on chromosome 17, and C) at the SC locus on 17q11.2.

Supplemental Figure S8. Summary of phecodes used in PheWAS on All of Us cohort. A) The number of recurrences per unique phecode. B) The total number of clinical codes exhibited per donor.

Supplemental Figure S9. Manhattan Plots for the *TFG-ADGRG7* PheWAS on All of Us cohort. A) Within the entire cohort, assuming a dominant disease model. B) Within the entire cohort, assuming an additive disease model. C) Within individuals with predicted European heritage only, assuming a dominant disease model. D) Within individuals with predicted European heritage only, assuming an additive disease model.

Supplemental Figure S10. Manhattan Plots for the *TRPM4-PPFIA3* PheWAS on All of Us cohort. A) Within the entire cohort, assuming a dominant disease model. B) Within the entire cohort, assuming an additive disease model. C) Within individuals with predicted African heritage only, assuming a dominant disease model. D) Within individuals with predicted African heritage only, assuming an additive disease model.

Supplementary Figure S11. IGV images of 28 SVs identified within this study, labeled with the chimeric RNA(s) that they generate.
